# Supplementary material for: Refined cell transfer model reveals roles for Ascl2 and Cxcr3 in splenic localization of mouse NK cells during virus infection
Source: J Immunol. 2025 Jul 22;214(8):1917–25. doi: 10.1093/jimmun/vkaf122 (PMC12313101; doi:10.1093/jimmun/vkaf122)
Supplement: vkaf122_Supplementary_Data [file vkaf122_supplementary_data.zip › JIMMUN-24-00315-s01.pdf]

Taconic Ms Genome Scan assessment of C57BL/6 background of Ascl2-floxed mouse
96.24% C57BL/6

Index	SNP Name	Chromosome	Position	C57BL/6J-1_GM SNP Ref Sample.GType	Ascl2 floxed mouse Genotype Result	Ascl2 floxed mouse Congenic Result
1217	CH1MM1	1	3267624	AA	AA	1
1770	CH1MM2	1	3562368	AA	AA	1
313	CH1MM3	1	4596262	AA	AA	1
1933	CH1MM4	1	5672259	AA	AA	1
314	CH1MM5	1	5976634	AA	AA	1
315	CH1MM6GM1	1	6726188	AA	AA	1
1222	CH1MM7	1	8153622	BB	BB	1
1411	CH1MM8	1	9094058	BB	BB	1
1761	CH1MM9	1	11097245	BB	BB	1
1452	CH1MM10	1	11339578	AA	AA	1
316	CH1MM11	1	12952339	BB	BB	1
1148	CH1MM12	1	16348817	AA	AA	1
317	CH1MM13	1	17378106	BB	BB	1
1067	CH1MM15	1	19361935	BB	BB	1
318	CH1MM16	1	19543954	AA	AA	1
1918	CH1MM17	1	20854452	BB	BB	1
249	CH1MM14	1	22238579	AA	AA	1
1669	CH1MM18	1	22780080	AA	AA	1
262	CH1MM19	1	25266300	AA	AA	1
1665	CH1MM20	1	25930912	AA	AA	1
1188	CH1MM21	1	27303342	AA	AA	1
319	CH1MM23	1	28340483	BB	BB	1
10	CH1MM22	1	28498625	AA	AA	1
1276	CH1MM24	1	29756328	AA	AA	1
320	CH1MM25	1	29790169	AA	AA	1
1598	CH1MM26	1	30069648	AA	AA	1
1354	CH1MM28	1	32550531	BB	BB	1
1205	CH1MM29	1	34170140	AA	AA	1
1218	CH1MM30	1	35918004	BB	BB	1
1593	CH1MM31	1	38029601	BB	BB	1
1552	CH1MM32	1	39035238	BB	BB	1
141	CH1MM33	1	40012957	BB	BB	1
321	CH1MM34	1	40973254	BB	BB	1
142	CH1MM35	1	41133737	BB	BB	1
322	CH1MM36	1	42503256	AA	AA	1
931	CH1MM37	1	44040278	BB	BB	1
27	CH1MM38	1	44364078	AA	AA	1
323	CH1MM39	1	45951485	AA	AA	1
1015	CH1MM40BLK4	1	46972003	AA	AA	1
324	CH1MM41	1	47520378	AA	AA	1
28	CH1MM42	1	49478483	BB	BB	1
29	CH1MM43	1	49662637	BB	BB	1
1463	CH1MM44	1	50279154	BB	BB	1
325	CH1MM45	1	51437808	BB	BB	1
326	CH1MM46	1	51825457	AA	AA	1
1674	CH1MM48	1	53515437	BB	BB	1
1891	CH1MM49	1	54134832	AA	AA	1
1453	CH1MM50	1	55594368	BB	BB	1
327	CH1MM52	1	58421667	AA	AA	1
1613	CH1MM51	1	58477200	BB	BB	1
1701	CH1MM53	1	59259617	AA	AA	1
1845	CH1MM54	1	59762338	BB	BB	1
1415	CH1MM55	1	61589987	AA	AA	1
1035	CH1MM56	1	62601861	AA	AA	1
1822	CH1MM57	1	63296244	BB	AA	0
11	CH1MM58	1	64791504	BB	BB	1
1902	CH1MM59	1	64912283	BB	BB	1
328	CH1MM60	1	66454071	AA	AA	1
329	CH1MM62	1	68750762	BB	AA	0
1809	CH1MM63	1	69041041	AA	BB	0
1683	CH1MM64	1	71993321	AA	AA	1
1398	CH1MM66	1	74480723	AA	AA	1

Symbol	Definition
AA	Homozygous for Allele1
AB	Heterozygous 1/2
BB	Homozygous for Allele2
1	Homozygous for C57BL/6J SNP
0.5	Heterozygous for C57BL/6J SNP and non-C57BL/6J SNP
0	Homozygous for non-C57BL/6J SNP
NC=no call	

1849	CH1MM67	1	74941196	AA	AA	1
330	CH1MM68	1	76351654	AA	BB	0
252	CH1MM69	1	76504841	BB	AA	0
143	CH1MM70	1	77881217	BB	AA	0
331	CH1MM71	1	78665382	BB	AA	0
246	CH1MM72	1	80303521	BB	BB	1
332	CH1MM73	1	80700423	BB	AA	0
144	CH1MM75	1	82127376	AA	BB	0
974	CH1MM74	1	82629293	AA	AA	1
145	CH1MM76	1	85709269	AA	BB	0
1454	CH1MM77	1	85909566	BB	AA	0
1949	CH1MM78	1	87118199	BB	BB	1
1690	CH1MM79	1	88510296	AA	AA	1
333	CH1MM80	1	91581068	BB	BB	1
1769	CH1MM81	1	93310260	AA	AA	1
1655	CH1MM82	1	95547258	AA	AA	1
1607	CH1MM83	1	95553643	AA	AA	1
1481	CH1MM84	1	96925219	BB	BB	1
334	CH1MM85	1	97713445	AA	AA	1
335	CH1MM86	1	99832586	AA	AA	1
1863	CH1MM87	1	101777460	BB	BB	1
23	CH1MM88	1	103278013	BB	BB	1
336	CH1MM89	1	103483879	AA	AA	1
1118	CH1MM90	1	104479609	BB	BB	1
1630	CH1MM91	1	105262861	BB	BB	1
24	CH1MM92	1	105429378	BB	BB	1
337	CH1MM93	1	106786462	AA	AA	1
1227	CH1MM94	1	109559424	BB	BB	1
338	CH1MM95	1	110539445	AA	AA	1
1986	CH1MM133	1	111120720	AA	NC	NA
1475	CH1MM96	1	111685307	AA	AA	1
339	CH1MM97	1	113789633	AA	AA	1
1923	CH1MM98	1	115351915	BB	BB	1
254	CH1MM99	1	115657934	BB	BB	1
1168	CH1MM100	1	117277713	BB	BB	1
1273	CH1MM101	1	118668165	BB	BB	1
1193	CH1MM102	1	120535176	BB	BB	1
340	CH1MM103GM3	1	122151792	BB	BB	1
1418	CH1MM104	1	122928345	AA	AA	1
1265	CH1MM105	1	123772836	BB	BB	1
1490	CH1MM106	1	125572446	BB	BB	1
341	CH1MM107	1	126495944	AA	AA	1
1725	CH1MM108	1	126554949	BB	BB	1
342	CH1MM109	1	128186670	BB	BB	1
1305	CH1MM111	1	131019919	BB	BB	1
1468	CH1MM112	1	131433134	AA	AA	1
343	CH1MM113	1	134138402	AA	AA	1
1943	CH1MM114	1	135083613	AA	AA	1
1782	CH1MM115	1	137306182	AA	AA	1
1752	CH1MM116	1	137568145	AA	AA	1
275	CH1MM47GM2-BM	1	138039993	AA	AA	1
344	CH1MM118	1	139159275	BB	BB	1
134	CH1MM119BLK8	1	139164775	BB	BB	1
345	CH1MM120	1	140461531	AA	AB	0.5
1882	CH1MM121	1	142449238	AA	AB	0.5
346	CH1MM122BLK9	1	142893540	BB	AB	0.5
347	CH1MM123	1	143988348	AA	AB	0.5
1602	CH1MM124	1	144571220	AA	AA	1
1158	CH1MM125	1	145057098	BB	AB	0.5
348	CH1MM126	1	146746254	AA	AA	1
1928	CH1MM127	1	148855669	BB	AB	0.5
349	CH1MM128	1	150519629	AA	AB	0.5
1899	CH1MM129	1	150900717	BB	BB	1
25	CH1MM130	1	152656727	BB	BB	1
1037	CH1MM132GM4	1	152789902	BB	AB	0.5
350	CH1MM131	1	154081181	BB	BB	1
1186	CH1MM134	1	156288854	AA	AB	0.5
1430	CH1MM135	1	156985575	BB	BB	1
1893	CH1MM136	1	157727955	AA	AA	1
1259	CH1MM137	1	160112660	BB	BB	1
351	CH1MM138	1	161853253	AA	AA	1
1226	CH1MM139	1	162077631	AA	AA	1
1987	CH1MM140	1	163824763	BB	BB	1
1352	CH1MM141	1	164219391	BB	BB	1
352	CH1MM142	1	166075636	BB	BB	1
1908	CH1MM143	1	167090282	BB	BB	1
1953	CH1MM144	1	167195376	BB	BB	1
1957	CH1MM146	1	171052494	BB	BB	1
1940	CH1MM147	1	171068538	BB	BB	1

310	CH1MM148	1	171962812	AA	AA	1
1356	CH1MM149	1	172318190	BB	AB	0.5
353	CH1MM150	1	173979189	BB	AB	0.5
1134	CH1MM151	1	174368713	AA	AB	0.5
1316	CH1MM152	1	175843083	AA	AA	1
354	CH1MM153	1	177097107	AA	AA	1
26	CH1MM154	1	178293310	BB	BB	1
355	CH1MM155	1	179438040	BB	AB	0.5
312	CH1MM156	1	180428677	AA	AA	1
356	CH1MM157	1	181244836	BB	BB	1
357	CH1MM158	1	181918031	BB	AB	0.5
1985	CH1MM159	1	182338146	BB	BB	1
1656	CH1MM160	1	183294052	BB	BB	1
233	CH1MM161	1	186616895	AA	AA	1
358	CH1MM163	1	190030771	BB	BB	1
1247	CH1MM164	1	192184304	AA	AA	1
906	CH1MM165	1	192540033	BB	BB	1
905	CH1MM166	1	192575644	AA	AA	1
914	CH1MM167	1	192816983	AA	AA	1
359	CH1MM168	1	193843898	BB	BB	1
253	CH1MM169	1	194990750	BB	BB	1
1399	CH2MM1	2	3234941	BB	BB	1
1710	CH2MM2	2	4029647	BB	BB	1
146	CH2MM3	2	4253476	AA	AA	1
1487	CH2MM4	2	4315026	BB	BB	1
1191	CH2MM5	2	5663011	BB	BB	1
360	CH2MM6	2	6105899	BB	BB	1
1855	CH2MM7	2	6751810	AA	AA	1
361	CH2MM8GM1	2	7380119	AA	AA	1
147	CH2MM9	2	9834423	BB	BB	1
1741	CH2MM10	2	10983250	BB	BB	1
1667	CH2MM11	2	12636479	AA	AA	1
1789	CH2MM12	2	13723022	BB	BB	1
137	CH2MM13	2	15672669	BB	BB	1
1914	CH2MM14	2	17805190	BB	BB	1
969	CH2MM15BLK4	2	18128492	BB	BB	1
362	CH2MM16GM3	2	19285392	AA	AA	1
363	CH2MM17	2	19403726	AA	AA	1
991	CH2MM18	2	20376761	AA	AA	1
1664	CH2MM19	2	20763455	AA	AB	0.5
1917	CH2MM20	2	23381550	BB	BB	1
1786	CH2MM21	2	24425115	BB	BB	1
364	CH2MM22	2	25985483	AA	AA	1
365	CH2MM23	2	28179100	AA	AA	1
266	CH2MM24	2	30077840	BB	BB	1
242	CH2MM25	2	31169956	BB	BB	1
1677	CH2MM26	2	31590167	BB	BB	1
66	CH2MM27	2	32911915	BB	BB	1
148	CH2MM28	2	33884632	AA	AA	1
1763	CH2MM29	2	35734932	AA	AA	1
366	CH2MM30	2	38001131	BB	BB	1
367	CH2MM31GM4	2	39704513	BB	BB	1
368	CH2MM32	2	41174115	AA	AA	1
1798	CH2MM33	2	42964700	BB	BB	1
369	CH2MM35	2	45583189	AA	AA	1
1766	CH2MM36	2	47054575	AA	AA	1
1753	CH2MM37	2	48563457	AA	AA	1
67	CH2MM38	2	50535024	BB	BB	1
370	CH2MM39	2	50679828	BB	BB	1
1472	CH2MM40	2	51355708	AA	AA	1
371	CH2MM41	2	53073622	BB	BB	1
372	CH2MM42	2	54702556	AA	AA	1
1622	CH2MM43	2	56225815	AA	AA	1
1425	CH2MM44	2	56425829	AA	AA	1
1159	CH2MM45	2	58079533	BB	BB	1
373	CH2MM46	2	59848581	BB	BB	1
1553	CH2MM47	2	61630428	AA	AA	1
68	CH2MM48	2	63035262	AA	AA	1
374	CH2MM49	2	63403198	BB	BB	1
1717	CH2MM50	2	64949345	AA	AA	1
375	CH2MM51	2	66798195	AA	AA	1
1880	CH2MM52	2	68080032	AA	AA	1
1865	CH2MM53	2	68819391	AA	AA	1
1486	CH2MM54	2	69981825	BB	BB	1
1117	CH2MM55	2	71428908	BB	BB	1
376	CH2MM57	2	73125349	AA	AA	1
69	CH2MM58	2	73306856	AA	AA	1
377	CH2MM59	2	74712211	BB	BB	1
149	CH2MM60	2	77485298	BB	BB	1

70	CH2MM61	2	79173789	BB	BB	1
1449	CH2MM62	2	80446033	AA	AA	1
1129	CH2MM63	2	81936462	BB	BB	1
1975	CH2MM65	2	84597400	BB	BB	1
1976	CH2MM64	2	84602679	AA	AA	1
1246	CH2MM66	2	85509018	BB	BB	1
378	CH2MM67	2	87183435	AA	AA	1
1920	CH2MM68	2	88835608	BB	BB	1
951	CH2MM70	2	91462491	AA	AA	1
379	CH2MM71	2	92657106	AA	AA	1
1165	CH2MM72	2	94902257	AA	AA	1
380	CH2MM73	2	95866110	BB	BB	1
1815	CH2MM74	2	96724230	BB	BB	1
1651	CH2MM75BLK13	2	96873527	AA	AA	1
71	CH2MM76	2	98152854	BB	BB	1
1876	CH2MM77	2	99314437	BB	BB	1
1921	CH2MM78	2	100190274	AA	AA	1
381	CH2MM79	2	101153386	AA	AA	1
150	CH2MM80	2	103730229	AA	AA	1
1073	CH2MM81	2	104021803	AA	AA	1
382	CH2MM82	2	105128249	AA	AA	1
383	CH2MM83	2	107295433	AA	AA	1
384	CH2MM84	2	108334935	AA	AA	1
385	CH2MM85	2	111034302	AA	AA	1
63	CH2MM86	2	111920430	BB	BB	1
1492	CH2MM87	2	114210365	AA	AA	1
1299	CH2MM88	2	115720278	AA	AA	1
386	CH2MM89	2	117123279	BB	BB	1
1984	CH2MM90	2	118997223	AA	AA	1
1554	CH2MM91	2	120326870	BB	BB	1
1076	CH2MM92	2	120775347	AA	AA	1
1457	CH2MM93	2	122149064	AA	AA	1
1911	CH2MM95	2	125139643	AA	AA	1
1947	CH2MM96	2	125143808	AA	AA	1
387	CH2MM97	2	126983411	AA	AA	1
151	CH2MM98	2	127642668	BB	BB	1
1091	CH2MM99	2	129986685	AA	AA	1
1123	CH2MM100	2	130855830	BB	BB	1
1555	CH2MM101	2	131835405	BB	BB	1
388	CH2MM102	2	133703052	BB	BB	1
64	CH2MM103	2	135969826	BB	BB	1
1941	CH2MM104	2	136720323	BB	BB	1
1657	CH2MM105	2	139021540	BB	BB	1
389	CH2MM106	2	140648455	AA	AA	1
390	CH2MM108	2	142242647	BB	BB	1
935	CH2MM110	2	143619762	BB	BB	1
936	CH2MM109GM5	2	143620791	BB	BB	1
391	CH2MM111	2	144215523	BB	BB	1
1058	CH2MM112	2	144375248	AA	AA	1
1859	CH2MM113	2	145874260	AA	AA	1
1285	CH2MM114	2	147493834	BB	BB	1
1706	CH2MM115	2	147897193	AA	AA	1
392	CH2MM116	2	149577344	AA	AA	1
1747	CH2MM117	2	149704217	AA	AA	1
1750	CH2MM119	2	152420670	BB	BB	1
997	CH2MM120	2	152952934	BB	BB	1
1873	CH2MM121	2	153983205	AA	AA	1
393	CH2MM122	2	156109398	AA	AA	1
1687	CH2MM123	2	157472532	BB	BB	1
1269	CH2MM124	2	157847043	AA	AA	1
394	CH2MM125	2	159511697	BB	BB	1
1115	CH2MM126	2	161222143	AA	AA	1
1151	CH2MM127	2	162994459	BB	BB	1
395	CH2MM128	2	164149447	AA	AA	1
396	CH2MM129	2	165238205	AA	AA	1
1392	CH2MM130	2	165852083	AA	AA	1
397	CH2MM131	2	166153313	BB	BB	1
65	CH2MM132	2	168365490	AA	AA	1
1482	CH2MM133	2	169683901	BB	BB	1
398	CH2MM134	2	170430328	BB	BB	1
399	CH2MM135	2	171230950	BB	BB	1
400	CH2MM136	2	173936808	AA	AA	1
401	CH2MM137	2	174698168	AA	AA	1
402	CH2MM138	2	178269102	BB	BB	1
1679	CH2MM139	2	179164554	AA	AA	1
1196	CH2MM140	2	179767999	AA	AA	1
403	CH3MM1	3	3850429	BB	BB	1
404	CH3MM2	3	4427520	AA	AA	1
405	CH3MM3	3	5694183	AA	AA	1

406	CH3MM4	3	7744877	BB	BB	1
407	CH3MM5	3	9756419	BB	BB	1
981	CH3MM6	3	9853284	AA	AA	1
408	CH3MM7	3	11080907	BB	BB	1
1751	CH3MM8	3	12166026	AA	AA	1
409	CH3MM9	3	14952956	BB	BB	1
1081	CH3MM10	3	16504455	AA	AA	1
152	CH3MM11	3	18093870	AA	AA	1
255	CH3MM12	3	19317476	AA	AA	1
1595	CH3MM13	3	19801759	BB	BB	1
1183	CH3MM15GM1	3	21084302	BB	BB	1
1731	CH3MM14	3	21585572	AA	AA	1
1409	CH3MM16	3	26693639	AA	AA	1
410	CH3MM17	3	27655383	BB	BB	1
1086	CH3MM18	3	30382915	AA	AA	1
411	CH3MM20	3	31534677	BB	BB	1
153	CH3MM22	3	33367977	AA	AA	1
1556	CH3MM23	3	34119827	AA	AA	1
1866	CH3MM24	3	34189588	AA	AA	1
1748	CH3MM25	3	36363257	AA	AA	1
1774	CH3MM26	3	38944035	BB	BB	1
1825	CH3MM27	3	40086629	AA	AA	1
290	CH3MM28	3	41267700	BB	BB	1
1093	CH3MM29	3	41961202	AA	AA	1
412	CH3MM30	3	43480339	AA	AA	1
75	CH3MM31	3	45551480	BB	BB	1
76	CH3MM32	3	46508489	AA	AA	1
413	CH3MM33	3	48282137	BB	BB	1
1629	CH3MM34	3	50415517	AA	AA	1
1499	CH3MM35	3	50557467	BB	BB	1
1465	CH3MM36	3	50710402	BB	BB	1
1742	CH3MM37	3	52497904	AA	AA	1
1833	CH3MM38	3	52925281	AA	AA	1
1557	CH3MM39	3	54956712	AA	AA	1
414	CH3MM40	3	56549657	AA	AA	1
1803	CH3MM42	3	58397291	AA	AA	1
1250	CH3MM43	3	59733145	AA	AA	1
415	CH3MM44	3	59784082	BB	BB	1
1736	CH3MM45	3	60418359	AA	AA	1
416	CH3MM46	3	60848155	AA	AA	1
417	CH3MM47	3	63547361	BB	BB	1
1709	CH3MM48	3	64893331	BB	BB	1
154	CH3MM49	3	66592222	BB	BB	1
418	CH3MM50	3	69652137	AA	AA	1
1696	CH3MM51	3	69923184	AA	AA	1
1764	CH3MM52	3	71424184	BB	BB	1
419	CH3MM53	3	75359033	BB	BB	1
1648	CH3MM54	3	76187876	AA	AA	1
420	CH3MM55	3	77158981	AA	AA	1
421	CH3MM56	3	78607165	AA	AA	1
1214	CH3MM57	3	79108200	BB	BB	1
422	CH3MM58	3	79835222	AA	AA	1
423	CH3MM59	3	80769015	AA	AA	1
424	CH3MM60	3	81267534	AA	AA	1
425	CH3MM61	3	83386588	BB	BB	1
1435	CH3MM62	3	85325744	AA	AA	1
1238	CH3MM64	3	86280013	AA	AA	1
1603	CH3MM63	3	86301613	AA	AA	1
1872	CH3MM65	3	86568504	BB	BB	1
1495	CH3MM66	3	87549833	AA	AA	1
426	CH3MM68	3	88761937	BB	BB	1
1960	CH3MM67	3	89136944	BB	BB	1
1708	CH3MM69	3	90129168	BB	BB	1
267	CH3MM70	3	90567921	AA	AA	1
427	CH3MM71	3	92026252	AA	AA	1
428	CH3MM72	3	93242062	AA	AA	1
930	CH3MM73	3	95366270	BB	BB	1
1948	CH3MM74	3	95398328	BB	BB	1
429	CH3MM75	3	95591406	AA	AA	1
1639	CH3MM76	3	95734004	BB	BB	1
1000	CH3MM77	3	97186615	AA	AA	1
1360	CH3MM79GM4	3	98866550	BB	BB	1
1478	CH3MM78	3	98938778	AA	AA	1
1031	CH3MM80	3	100627011	BB	BB	1
1496	CH3MM81	3	102596481	AA	AA	1
1322	CH3MM82	3	103541444	AA	AA	1
430	CH3MM83	3	105377721	BB	BB	1
1326	CH3MM84	3	107036460	AA	AA	1
431	CH3MM85	3	109099723	BB	BB	1

1178	CH3MM86	3	110163628	AA	AA	1
1296	CH3MM87	3	112141148	BB	BB	1
1375	CH3MM88	3	115447022	BB	BB	1
155	CH3MM89	3	116336508	AA	AA	1
432	CH3MM90	3	116696730	BB	BB	1
1367	CH3MM91	3	116789332	BB	BB	1
1362	CH3MM92	3	117447888	AA	AA	1
433	CH3MM94	3	118652326	AA	AA	1
1633	CH3MM93BLK11	3	118901968	AA	AA	1
72	CH3MM95	3	119813532	BB	BB	1
1079	CH3MM96	3	121413685	AA	AA	1
921	CH3MM97	3	122055383	AA	AA	1
920	CH3MM98	3	122059991	BB	BB	1
434	CH3MM99	3	122631307	BB	BB	1
247	CH3MM100	3	123167779	BB	BB	1
435	CH3MM101	3	124904845	AA	AA	1
1146	CH3MM102	3	126207312	AA	AA	1
1647	CH3MM103	3	127732396	BB	BB	1
1140	CH3MM104	3	128086184	BB	BB	1
436	CH3MM105	3	130163596	AA	BB	0
437	CH3MM106	3	131344630	BB	BB	1
73	CH3MM107	3	132231864	AA	AA	1
438	CH3MM108	3	133947914	BB	BB	1
439	CH3MM110	3	134638137	AA	AA	1
1631	CH3MM109	3	134676184	BB	BB	1
1172	CH3MM111	3	135923372	BB	BB	1
440	CH3MM112	3	137772028	BB	BB	1
441	CH3MM113	3	139659962	BB	BB	1
1793	CH3MM115	3	142340602	BB	BB	1
1922	CH3MM116	3	142763641	BB	BB	1
1376	CH3MM118	3	145975081	AA	AA	1
1063	CH3MM119	3	148071178	BB	BB	1
442	CH3MM121	3	149860748	BB	BB	1
1014	CH3MM120GM5	3	150492294	BB	BB	1
1466	CH3MM122	3	151039228	BB	BB	1
443	CH3MM123	3	151269908	BB	BB	1
444	CH3MM124	3	154030206	BB	BB	1
445	CH3MM125	3	154545243	AA	AA	1
156	CH3MM126	3	156845426	BB	BB	1
74	CH3MM127	3	158416544	AA	AA	1
1274	CH4MM1	4	3722678	AA	AA	1
157	CH4MM2	4	5086430	AA	AA	1
158	CH4MM3	4	5679199	AA	AA	1
446	CH4MM4GM1	4	6710941	BB	BB	1
1087	CH4MM5	4	7200289	BB	BB	1
447	CH4MM6GM2	4	8803714	AA	AA	1
1994	CH4MM7	4	10899090	BB	BB	1
1916	CH4MM8	4	11707170	BB	BB	1
448	CH4MM9	4	13837795	BB	BB	1
236	CH4MM10	4	15394345	AA	AA	1
1788	CH4MM11	4	18118163	BB	BB	1
77	CH4MM12	4	19581155	AA	AA	1
449	CH4MM13	4	20739624	BB	BB	1
159	CH4MM14	4	22379321	AA	AA	1
1046	CH4MM15	4	22958388	BB	BB	1
1605	CH4MM16	4	23496605	BB	BB	1
235	CH4MM17	4	24223752	AA	AA	1
450	CH4MM18	4	26959191	AA	AA	1
1319	CH4MM19	4	29263187	AA	AA	1
78	CH4MM20	4	30421807	AA	AA	1
1109	CH4MM21	4	30984142	BB	BB	1
1038	CH4MM22	4	31909848	AA	AA	1
451	CH4MM24	4	34982749	BB	BB	1
1433	CH4MM25	4	36129678	BB	BB	1
1220	CH4MM26	4	38322870	AA	AA	1
452	CH4MM27	4	39790899	AA	AA	1
79	CH4MM28	4	40449935	AA	AA	1
1494	CH4MM29	4	41657026	BB	BB	1
1293	CH4MM30	4	43184799	BB	BB	1
454	CH4MM31	4	44866546	AA	AA	1
455	CH4MM32	4	46175357	AA	AA	1
453	CH4MM33	4	46642823	BB	BB	1
1108	CH4MM34	4	48113335	AA	AA	1
1177	CH4MM35	4	51790315	BB	BB	1
456	CH4MM36	4	53434354	AA	AA	1
1065	CH4MM37	4	54664413	AA	AA	1
1739	CH4MM39	4	56497135	AA	AA	1
457	CH4MM40	4	57762480	AA	AA	1
1391	CH4MM41	4	59835281	BB	BB	1

1033	CH4MM47BLK5	4	62373986	AA	AA	1
1249	CH4MM42	4	62625445	BB	BB	1
1	CH4MM43GM4	4	62641213	AA	AA	1
1871	CH4MM44	4	63294034	BB	BB	1
1754	CH4MM45	4	63603776	AA	AA	1
1829	CH4MM46	4	63816263	BB	BB	1
458	CH4MM48	4	66401880	AA	AA	1
904	CH4MM49	4	66807350	BB	BB	1
459	CH4MM50	4	69195334	AA	AA	1
1372	CH4MM51	4	70318346	AA	AA	1
460	CH4MM52	4	71143968	AA	AA	1
1716	CH4MM53	4	72058475	AA	AA	1
461	CH4MM54	4	73459299	AA	AA	1
462	CH4MM55	4	76047252	BB	BB	1
1363	CH4MM56	4	76788915	AA	AA	1
80	CH4MM57	4	79596055	AA	AA	1
278	CH4MM60GM5-BM	4	80753589	BB	BB	1
1357	CH4MM58	4	81222410	AA	AA	1
1497	CH4MM59	4	81613324	BB	BB	1
1180	CH4MM61	4	82683420	BB	BB	1
1300	CH4MM62	4	83106699	AA	AA	1
463	CH4MM63	4	84028170	AA	AB	0.5
1384	CH4MM64	4	86149979	AA	AA	1
464	CH4MM65	4	88869918	AA	AA	1
1705	CH4MM66	4	89827869	BB	BB	1
465	CH4MM67	4	91009503	BB	AB	0.5
1771	CH4MM68	4	92866645	AA	AA	1
1078	CH4MM69	4	95836099	BB	BB	1
466	CH4MM70	4	97232474	AA	AA	1
1336	CH4MM71	4	98837414	AA	AB	0.5
467	CH4MM72	4	99208267	AA	AB	0.5
274	CH4MM75GM6-BM	4	101668602	BB	BB	1
1459	CH4MM76	4	103169169	AA	AB	0.5
468	CH4MM77	4	103819210	BB	AB	0.5
1824	CH4MM78	4	105835311	AA	AB	0.5
1723	CH4MM79	4	108568003	BB	AA	0
1377	CH4MM80	4	109892274	AA	BB	0
1368	CH4MM81	4	111479888	BB	BB	1
1878	CH4MM82	4	115893191	BB	AA	0
1257	CH4MM83	4	118249246	BB	AA	0
1862	CH4MM84	4	119038254	AA	BB	0
1241	CH4MM85	4	119578605	AA	AA	1
1169	CH4MM86	4	120916042	BB	AA	0
469	CH4MM87	4	122845628	AA	AA	1
470	CH4MM88	4	124089747	BB	AA	0
1405	CH4MM89	4	124101664	BB	AA	0
992	CH4MM91BLK11	4	125062493	BB	BB	1
471	CH4MM90	4	126605900	BB	BB	1
160	CH4MM92	4	127923842	BB	BB	1
1848	CH4MM93	4	129607118	AA	BB	0
472	CH4MM94	4	129847275	BB	BB	1
1995	CH4MM95	4	132979273	AA	BB	0
1111	CH4MM96	4	135456793	AA	AA	1
473	CH4MM97	4	136401961	BB	BB	1
1026	CH4MM99	4	138471174	AA	AA	1
1890	CH4MM98	4	139495154	AA	AA	1
1237	CH4MM100	4	141297399	AA	BB	0
1440	CH4MM101	4	142231852	AA	AA	1
1001	CH4MM102	4	142908937	AA	BB	0
1728	CH4MM103	4	147984211	AA	BB	0
1768	CH4MM104	4	152100523	AA	AA	1
1641	CH4MM108	4	153590104	BB	BB	1
476	CH4MM105	4	154514465	BB	BB	1
474	CH4MM106	4	155302630	AA	AA	1
475	CH4MM107	4	155587033	AA	AA	1
1780	CH4MM109	4	156268185	AA	AA	1
477	CH5MM2	5	3645358	AA	AA	1
478	CH5MM3GM1	5	4018725	AA	AA	1
479	CH5MM4	5	4354103	BB	BB	1
1116	CH5MM5	5	4518201	AA	AA	1
84	CH5MM6	5	4647869	AA	AA	1
1915	CH5MM7	5	5482079	BB	BB	1
1745	CH5MM8GM2	5	7511341	BB	BB	1
1806	CH5MM9	5	7694169	AA	AA	1
480	CH5MM10	5	9791229	AA	AA	1
1403	CH5MM11	5	12421074	BB	BB	1
1234	CH5MM12	5	14374078	BB	BB	1
1843	CH5MM13	5	14855076	AA	AA	1
1253	CH5MM14	5	18065828	AA	AA	1

1585	CH5MM16	5	19327638	AA	AA	1
1730	CH5MM15	5	19507121	AA	AA	1
481	CH5MM17	5	21819245	AA	AA	1
482	CH5MM18	5	23219131	AA	AA	1
1842	CH5MM19	5	25076638	AA	AA	1
1345	CH5MM20	5	25320971	AA	AA	1
82	CH5MM21	5	26002924	BB	BB	1
483	CH5MM22	5	27319349	AA	AA	1
933	CH5MM28GM3	5	27660023	BB	BB	1
932	CH5MM26	5	27665780	BB	BB	1
1283	CH5MM23	5	27914471	AA	AA	1
924	CH5MM27	5	27925996	BB	BB	1
1658	CH5MM24	5	28622180	AA	AA	1
1200	CH5MM25	5	28967974	BB	BB	1
311	CH5MM29	5	31235209	AA	AA	1
1313	CH5MM30	5	32821767	AA	AA	1
1071	CH5MM31	5	33474844	AA	AA	1
291	CH5MM32	5	35066121	AA	AA	1
1163	CH5MM33	5	35676268	AA	AA	1
484	CH5MM34	5	37164964	BB	BB	1
1836	CH5MM35	5	37258905	BB	BB	1
1617	CH5MM36	5	38417980	AA	AA	1
1756	CH5MM37	5	39921797	AA	AA	1
485	CH5MM38	5	41200017	AA	AA	1
1713	CH5MM39	5	44460961	BB	BB	1
486	CH5MM40	5	45128712	AA	AA	1
487	CH5MM41	5	45513640	BB	BB	1
1080	CH5MM42	5	46236764	BB	BB	1
83	CH5MM43	5	47405099	BB	BB	1
1686	CH5MM44	5	49503838	AA	AA	1
1604	CH5MM46	5	50546308	AA	AA	1
1400	CH5MM45	5	50853413	AA	AA	1
1088	CH5MM47	5	52555329	BB	BB	1
1113	CH5MM48	5	53814423	AA	AA	1
241	CH5MM49	5	55381352	BB	BB	1
85	CH5MM50	5	57690018	AA	AA	1
1042	CH5MM51BLK5	5	58234182	BB	BB	1
1025	CH5MM52GM4	5	60389802	BB	BB	1
1846	CH5MM53	5	61663730	AA	AA	1
1678	CH5MM54	5	62959403	AA	AA	1
243	CH5MM55	5	64346999	AA	AA	1
1559	CH5MM56	5	66819168	BB	BB	1
1558	CH5MM57	5	66819410	AA	AA	1
488	CH5MM58	5	67304413	BB	BB	1
1268	CH5MM60	5	70571935	BB	BB	1
1835	CH5MM61	5	72954333	BB	BB	1
1660	CH5MM62	5	74555925	AA	AA	1
161	CH5MM63	5	75039329	BB	BB	1
237	CH5MM64	5	76479803	BB	BB	1
489	CH5MM65	5	77593401	BB	BB	1
86	CH5MM66	5	80435765	BB	BB	1
490	CH5MM67	5	81278356	BB	BB	1
1131	CH5MM69	5	83189840	AA	AA	1
292	CH5MM70	5	87239940	AA	AA	1
87	CH5MM71	5	88543679	AA	AA	1
162	CH5MM72	5	89700468	AA	AA	1
1162	CH5MM73	5	89835553	BB	BB	1
491	CH5MM74	5	91007807	AA	AA	1
1040	CH5MM76	5	92228938	AA	AA	1
1729	CH5MM75	5	92258778	AA	AA	1
492	CH5MM77	5	96338460	BB	BB	1
88	CH5MM78	5	96488572	BB	BB	1
89	CH5MM79	5	96786637	BB	BB	1
1090	CH5MM80	5	97985215	BB	BB	1
1337	CH5MM81	5	99448665	BB	BB	1
493	CH5MM82	5	104076830	BB	BB	1
1479	CH5MM83	5	108532530	AA	AA	1
283	CH5MM86GM5-BM	5	108569081	BB	BB	1
494	CH5MM84	5	108802578	AA	AA	1
306	CH5MM85	5	110253056	BB	BB	1
1997	CH5MM87	5	111497312	BB	BB	1
163	CH5MM88	5	112221528	AA	AA	1
495	CH5MM89	5	114348999	AA	AA	1
164	CH5MM90	5	116244517	AA	AA	1
1105	CH5MM91	5	116892854	AA	AA	1
1097	CH5MM92BLK10	5	117597413	AA	AA	1
496	CH5MM93	5	118093674	BB	BB	1
1597	CH5MM96	5	119252582	BB	BB	1
81	CH5MM94	5	120026195	BB	BB	1

1934	CH5MM95	5	121705940	AA	AA	1
497	CH5MM97	5	122371608	AA	AA	1
498	CH5MM98	5	123929067	BB	BB	1
1147	CH5MM99	5	124886863	AA	AA	1
165	CH5MM100	5	126190573	BB	BB	1
166	CH5MM101	5	126636604	BB	BB	1
499	CH5MM102	5	127861619	AA	AA	1
1242	CH5MM105GM6	5	128882625	AA	AA	1
500	CH5MM103	5	129335949	AA	AA	1
167	CH5MM104	5	130401982	BB	BB	1
501	CH5MM106	5	134073856	AA	AA	1
909	CH5MM108	5	134324450	AA	AA	1
910	CH5MM109	5	134432495	AA	AA	1
899	CH5MM110	5	134642114	AA	AA	1
900	CH5MM111	5	134688366	BB	BB	1
911	CH5MM112	5	134806600	AA	AA	1
901	CH5MM113	5	134986971	BB	BB	1
902	CH5MM114	5	135137836	BB	BB	1
912	CH5MM115	5	135159440	AA	AA	1
903	CH5MM116	5	135327065	BB	BB	1
913	CH5MM117	5	135670753	AA	AA	1
502	CH5MM107	5	136562415	BB	BB	1
980	CH5MM120	5	137161777	AA	AA	1
1560	CH5MM118	5	138455352	AA	AA	1
503	CH5MM119	5	139480440	BB	BB	1
1801	CH5MM121	5	139750409	AA	AA	1
1060	CH5MM122	5	141767922	BB	BB	1
504	CH5MM123	5	144044303	AA	AA	1
1998	CH5MM124	5	144493250	AA	AA	1
505	CH5MM125	5	145155288	AA	AA	1
1428	CH5MM126	5	149514118	BB	BB	1
917	CH5MM127	5	149910533	AA	AA	1
907	CH5MM128	5	150219582	BB	BB	1
916	CH5MM129	5	150220556	BB	BB	1
915	CH5MM130	5	150237410	AA	AA	1
1095	CH6MM1	6	3466871	BB	BB	1
506	CH6MM2	6	3847194	BB	BB	1
507	CH6MM3GM1	6	4459446	AA	AA	1
1668	CH6MM4	6	4657980	AA	AA	1
1637	CH6MM5	6	5897944	AA	AA	1
1307	CH6MM6	6	6435778	AA	AA	1
508	CH6MM7GM2	6	8014243	AA	AA	1
509	CH6MM8GM3	6	9181676	AA	AA	1
510	CH6MM9	6	10160651	AA	AA	1
511	CH6MM10	6	11276255	AA	AA	1
1644	CH6MM11	6	13947255	AA	AA	1
1194	CH6MM12	6	14289792	AA	AA	1
512	CH6MM13	6	16061005	BB	BB	1
1053	CH6MM14	6	17972567	BB	BB	1
513	CH6MM15	6	18568251	AA	AA	1
514	CH6MM16	6	21943927	BB	BB	1
1223	CH6MM17	6	24415693	BB	BB	1
1150	CH6MM18	6	25814203	BB	BB	1
515	CH6MM19	6	27951181	AA	AA	1
12	CH6MM20BLK2	6	28715913	BB	BB	1
273	CH6MM21GM4-BM	6	29070991	BB	BB	1
168	CH6MM22	6	29410902	BB	BB	1
516	CH6MM23	6	32827586	AA	AA	1
517	CH6MM24	6	34484392	BB	BB	1
1332	CH6MM25	6	36094017	BB	BB	1
1832	CH6MM26	6	37508711	AA	AA	1
518	CH6MM27	6	37666464	BB	BB	1
519	CH6MM28	6	38522224	BB	BB	1
169	CH6MM29	6	40776231	BB	BB	1
520	CH6MM30	6	43803697	AA	AA	1
521	CH6MM31	6	44172660	BB	BB	1
522	CH6MM32	6	44514263	BB	BB	1
1734	CH6MM33	6	45223600	AA	AA	1
523	CH6MM34	6	45410336	AA	AA	1
524	CH6MM35	6	49019686	BB	BB	1
1646	CH6MM36	6	49583193	AA	AA	1
1498	CH6MM37	6	49846526	AA	AA	1
525	CH6MM38	6	51174292	AA	AA	1
526	CH6MM39	6	53728674	BB	BB	1
1847	CH6MM40	6	56333806	BB	BB	1
998	CH6MM41GM5	6	56545382	AA	AA	1
91	CH6MM42	6	57086979	BB	BB	1
527	CH6MM43	6	59500337	BB	BB	1
1877	CH6MM44	6	63800018	AA	AA	1

528	CH6MM45	6	65084735	AA	AA	1
1844	CH6MM46	6	67412672	AA	AA	1
1691	CH6MM47	6	69716615	BB	BB	1
1484	CH6MM48	6	71111444	AA	AA	1
272	CH6MM49	6	71305186	AA	AA	1
529	CH6MM50	6	73285195	BB	BB	1
1152	CH6MM51	6	75372604	BB	BB	1
1302	CH6MM52	6	76416547	AA	AA	1
1339	CH6MM53	6	77078807	BB	BB	1
530	CH6MM54	6	78215907	BB	BB	1
1874	CH6MM55	6	79786668	AA	AA	1
1614	CH6MM56BLK10	6	80282672	AA	AA	1
1672	CH6MM57	6	81426039	AA	AA	1
92	CH6MM58	6	83174307	AA	AA	1
293	CH6MM59	6	84867285	AA	AA	1
93	CH6MM60	6	85937947	BB	BB	1
1767	CH6MM61	6	86858731	AA	AA	1
531	CH6MM62	6	89641965	AA	AA	1
1588	CH6MM63	6	91752018	BB	BB	1
532	CH6MM65	6	91988037	AA	AA	1
968	CH6MM64	6	92140030	AA	AA	1
1784	CH6MM66	6	93512957	BB	BB	1
1735	CH6MM67	6	94032928	BB	BB	1
533	CH6MM68	6	95360331	AA	AA	1
1841	CH6MM69	6	96724515	BB	BB	1
534	CH6MM70	6	97904993	AA	AA	1
1493	CH6MM71	6	98390487	AA	AA	1
1427	CH6MM72	6	101099475	AA	AA	1
535	CH6MM73	6	103727177	AA	AA	1
1704	CH6MM74	6	104865850	BB	BB	1
1609	CH6MM75	6	105560958	AA	AA	1
536	CH6MM76	6	106282414	BB	BB	1
1142	CH6MM77	6	109929101	AA	AA	1
289	CH6MM78	6	110717193	AA	AA	1
537	CH6MM79	6	111276755	BB	BB	1
538	CH6MM80	6	112501423	AA	AA	1
1900	CH6MM81	6	114384207	BB	BB	1
1912	CH6MM82	6	116067234	AA	AA	1
238	CH6MM83	6	116733640	AA	AA	1
539	CH6MM84	6	118049388	AA	AA	1
1275	CH6MM85	6	119984112	AA	AA	1
1029	CH6MM86	6	120555770	BB	BB	1
265	CH6MM87	6	121276268	AA	AA	1
170	CH6MM88	6	124033403	BB	BB	1
540	CH6MM89	6	125025551	AA	AA	1
1698	CH6MM90	6	125694448	AA	AA	1
1002	CH6MM91	6	127850866	AA	AA	1
1204	CH6MM92	6	127981496	AA	AA	1
90	CH6MM93	6	130215123	AA	AA	1
1450	CH6MM94	6	131285221	BB	BB	1
1330	CH6MM95	6	131967416	BB	BB	1
1834	CH6MM96	6	133944697	AA	AA	1
541	CH6MM97	6	134228198	AA	AA	1
1003	CH6MM98	6	135359886	BB	BB	1
542	CH6MM99	6	136367262	BB	BB	1
966	CH6MM100	6	137513153	AA	AA	1
965	CH6MM101	6	137513315	AA	AA	1
949	CH6MM102	6	137526591	AA	AA	1
950	CH6MM103	6	137527369	AA	AA	1
959	CH6MM104	6	137557654	AA	AA	1
947	CH6MM105	6	137565491	AA	AA	1
948	CH6MM106	6	137567504	AA	AA	1
964	CH6MM107	6	137571408	AA	AA	1
946	CH6MM108	6	137577035	AA	AA	1
945	CH6MM109	6	137577248	AA	AA	1
963	CH6MM110	6	137595315	AA	AA	1
957	CH6MM111	6	137606332	BB	BB	1
955	CH6MM112	6	137609838	BB	BB	1
958	CH6MM113	6	137609850	BB	BB	1
962	CH6MM114	6	137610694	BB	BB	1
956	CH6MM115	6	137612503	BB	BB	1
952	CH6MM116	6	137618045	BB	BB	1
953	CH6MM117	6	137618137	BB	BB	1
961	CH6MM118	6	137618665	AA	AA	1
960	CH6MM119	6	137618835	BB	BB	1
954	CH6MM120	6	137626612	AA	AA	1
543	CH6MM121	6	138231959	BB	BB	1
1160	CH6MM122	6	139802936	BB	BB	1
1831	CH6MM123	6	142366045	AA	AA	1

544	CH6MM124	6	143982499	BB	BB	1
1075	CH6MM125	6	144260903	AA	AA	1
1892	CH6MM126	6	145691056	AA	AA	1
545	CH6MM127GM6	6	145727333	AA	AA	1
1765	CH6MM128	6	147156305	AA	AA	1
1379	CH6MM129	6	148213396	AA	AA	1
171	CH6MM130	6	149167263	AA	AA	1
986	CH7MM2	7	3564400	BB	BB	1
1310	CH7MM46	7	4238895	AA	AA	1
2	CH7MM1GM1	7	4353873	AA	AA	1
1407	CH7MM12	7	4369780	AA	AA	1
1077	CH7MM27	7	6959792	BB	BB	1
250	CH7MM3	7	9897807	BB	BB	1
97	CH7MM4	7	11445733	BB	BB	1
1070	CH7MM44	7	12810738	AA	AA	1
1032	CH7MM11GM2	7	14342527	AA	AA	1
1171	CH7MM5	7	15770099	BB	BB	1
1464	CH7MM6	7	16005612	BB	BB	1
546	CH7MM7	7	16295858	AA	AA	1
1857	CH7MM8	7	17383241	AA	AA	1
1243	CH7MM9	7	18757737	BB	BB	1
172	CH7MM10	7	19136932	AA	AA	1
923	CH7MM23	7	23042415	BB	BB	1
1981	CH7MM24	7	23990442	BB	BB	1
547	CH7MM14	7	24637291	AA	AA	1
926	CH7MM28BLK2	7	26645536	AA	AA	1
95	CH7MM16	7	27854632	AA	AA	1
1561	CH7MM17	7	28531637	BB	BB	1
269	CH7MM18	7	28724942	BB	BB	1
1562	CH7MM19	7	29480878	BB	BB	1
942	CH7MM32	7	29887388	BB	BB	1
943	CH7MM33	7	29890105	AA	AA	1
927	CH7MM34	7	29891812	BB	BB	1
928	CH7MM35	7	29892197	BB	BB	1
1966	CH7MM36	7	30122780	BB	BB	1
1965	CH7MM37	7	30124310	BB	BB	1
1027	CH7MM20	7	30218063	AA	AA	1
1103	CH7MM38GM4	7	30422632	BB	BB	1
1004	CH7MM41	7	33333557	BB	BB	1
1434	CH7MM21	7	33932439	BB	BB	1
1737	CH7MM22	7	34398277	AA	AA	1
548	CH7MM25	7	35831560	BB	BB	1
1441	CH7MM26	7	36482915	AA	AA	1
1145	CH7MM29	7	40066481	BB	BB	1
173	CH7MM30	7	40574598	AA	AA	1
1584	CH7MM31	7	40975744	AA	AA	1
967	CH7MM50	7	43646851	BB	BB	1
1432	CH7MM39	7	44188021	AA	AA	1
1724	CH7MM40	7	44540550	AA	AA	1
1946	CH7MM53	7	45748966	AA	AA	1
1964	CH7MM42	7	46076833	AA	AA	1
96	CH7MM43	7	48932295	AA	AA	1
1606	CH7MM56	7	49629985	BB	BB	1
1308	CH7MM45	7	50668350	BB	BB	1
549	CH7MM47	7	52054651	BB	BB	1
1424	CH7MM48	7	53356646	AA	AA	1
1107	CH7MM49GM5	7	55782489	BB	BB	1
1277	CH7MM51	7	56756734	AA	AA	1
1034	CH7MM52GM6	7	57224049	AA	AA	1
1963	CH7MM62	7	57377095	AA	AA	1
550	CH7MM54	7	58359043	AA	AA	1
245	CH7MM55	7	60616808	AA	AA	1
1197	CH7MM57	7	64050033	AA	AA	1
1406	CH7MM58	7	64162533	BB	BB	1
1659	CH7MM59	7	65931037	BB	BB	1
1334	CH7MM60	7	68172469	BB	BB	1
551	CH7MM61	7	69446194	AA	AA	1
552	CH7MM63	7	70493640	AA	AA	1
553	CH7MM64	7	70947963	BB	BB	1
1175	CH7MM65	7	72336014	BB	BB	1
985	CH7MM74	7	72470482	AA	AA	1
554	CH7MM66	7	73708684	BB	BB	1
1187	CH7MM67	7	74279097	BB	BB	1
555	CH7MM68	7	75937371	BB	BB	1
556	CH7MM69	7	77936043	BB	BB	1
1611	CH7MM79	7	78906936	BB	BB	1
557	CH7MM70	7	79347967	AA	AA	1
558	CH7MM71	7	80787931	AA	AA	1
1711	CH7MM72	7	81931309	AA	AA	1

559	CH7MM73	7	83918212	AA	AA	1
560	CH7MM75	7	86783390	AA	AA	1
277	CH7MM92GM9-BM	7	87142202	AA	AA	1
561	CH7MM76	7	88348412	AA	AA	1
1074	CH7MM87GM7	7	89263322	BB	BB	1
562	CH7MM77	7	89709592	BB	BB	1
98	CH7MM78	7	90266736	AA	AA	1
563	CH7MM89GM8	7	91471709	AA	AA	1
1563	CH7MM80	7	92582114	BB	BB	1
1781	CH7MM81	7	93336778	AA	AA	1
564	CH7MM82	7	95152066	BB	BB	1
1298	CH7MM83	7	95751918	BB	BB	1
1587	CH7MM95	7	96427639	AA	AA	1
934	CH7MM96	7	98276092	BB	BB	1
1350	CH7MM84	7	98370170	BB	BB	1
565	CH7MM85	7	99677906	BB	BB	1
1396	CH7MM86	7	101418884	AA	AA	1
1850	CH7MM88	7	101890458	BB	BB	1
1164	CH7MM90	7	106675644	AA	AA	1
566	CH7MM91	7	106968243	AA	AA	1
1889	CH7MM93	7	107202061	BB	BB	1
567	CH7MM94	7	109057371	AA	AA	1
568	CH7MM97	7	111547760	BB	BB	1
1359	CH7MM98	7	111605831	BB	BB	1
1624	CH7MM109	7	113379033	BB	BB	1
1467	CH7MM99	7	113647449	BB	BB	1
1689	CH7MM100	7	113965297	BB	BB	1
569	CH7MM101	7	116837717	BB	BB	1
1778	CH7MM115GM11	7	117951219	AA	AA	1
1005	CH7MM102	7	118882336	AA	AA	1
258	CH7MM103	7	119554693	BB	BB	1
1369	CH7MM104	7	119727205	BB	BB	1
1600	CH7MM118	7	120293029	BB	BB	1
570	CH7MM106	7	122165763	AA	AA	1
1776	CH7MM107	7	123411251	BB	BB	1
571	CH7MM108	7	124278488	BB	BB	1
1206	CH7MM110	7	125939324	BB	BB	1
572	CH7MM111	7	126707285	BB	BB	1
573	CH7MM112	7	127589676	AA	AA	1
1621	CH7MM122	7	127990842	AA	AA	1
1036	CH7MM113GM10	7	128016550	BB	BB	1
574	CH7MM114BLK8	7	128637419	AA	AA	1
1962	CH7MM123	7	130580770	BB	BB	1
575	CH7MM116	7	131221533	BB	BB	1
1413	CH7MM117	7	132599886	AA	AA	1
94	CH7MM119	7	134730055	BB	BB	1
576	CH7MM120	7	136890313	AA	AA	1
1112	CH7MM121GM12	7	138899085	BB	AB	0.5
922	CH7MM125	7	141931204	AA	AA	1
1938	CH7MM126	7	144487580	AA	BB	0
1939	CH7MM127	7	144487962	BB	AA	0
1714	CH7MM124	7	144735438	BB	AA	0
1385	CH8MM1	8	4273543	AA	AA	1
1856	CH8MM2	8	6858351	BB	BB	1
1773	CH8MM3	8	7900108	AA	AA	1
1653	CH8MM4	8	8349969	AA	AA	1
1643	CH8MM5	8	8896657	BB	BB	1
577	CH8MM6	8	11717579	AA	AA	1
1925	CH8MM7	8	14351465	BB	BB	1
578	CH8MM8	8	15329372	BB	BB	1
579	CH8MM9	8	16283591	BB	BB	1
1066	CH8MM10	8	16586587	BB	BB	1
174	CH8MM11	8	17629135	BB	BB	1
264	CH0MM1	8	21413418	AA	AA	1
1500	CH8MM12	8	23022771	BB	BB	1
175	CH8MM13	8	23712096	AA	AA	1
1094	CH8MM14	8	23937260	BB	BB	1
13	CH8MM18BLK4	8	25875668	AA	AA	1
580	CH8MM15	8	26022438	AA	AA	1
1331	CH8MM16	8	26794789	AA	AA	1
99	CH8MM17	8	27665270	BB	BB	1
176	CH8MM19	8	28892546	AA	AA	1
581	CH8MM20	8	29724286	BB	AB	0.5
582	CH8MM21GM1	8	31188669	AA	AB	0.5
1564	CH8MM22	8	31751524	AA	AB	0.5
1348	CH8MM23	8	32355117	BB	AB	0.5
1263	CH8MM24	8	35014932	AA	AA	1
100	CH8MM25	8	35320502	AA	AA	1
1120	CH8MM26	8	36661507	AA	AB	0.5

1133	CH8MM27	8	38959180	BB	AB	0.5
583	CH8MM28	8	40162248	BB	BB	1
1329	CH8MM29	8	41109165	BB	AB	0.5
1125	CH8MM30	8	43417345	AA	AB	0.5
1887	CH8MM31	8	45265063	AA	AB	0.5
584	CH8MM32	8	46016896	AA	AB	0.5
1436	CH8MM33	8	50095356	BB	BB	1
585	CH8MM34	8	51005096	AA	AA	1
586	CH8MM35	8	51407251	AA	AA	1
101	CH8MM36	8	52361055	AA	AA	1
102	CH8MM37	8	52888323	BB	BB	1
1043	CH8MM39GM2	8	53013489	AA	AA	1
587	CH8MM38	8	54809329	BB	BB	1
588	CH8MM40	8	56831658	AA	AA	1
1471	CH8MM41	8	57809495	BB	BB	1
1224	CH8MM42	8	58216276	BB	BB	1
589	CH8MM43GM3	8	59214208	BB	BB	1
1353	CH8MM44	8	59403246	BB	BB	1
1157	CH8MM45	8	61674770	BB	BB	1
590	CH8MM46	8	62435245	AA	AA	1
1130	CH8MM47	8	64455732	AA	AA	1
1869	CH8MM48	8	64578178	AA	AA	1
591	CH8MM49	8	66070504	AA	AA	1
1594	CH8MM50	8	66273745	BB	BB	1
592	CH8MM51	8	67505959	AA	AA	1
1020	CH8MM53	8	68536215	AA	AA	1
1062	CH8MM52	8	68649444	AA	AA	1
1950	CH8MM55	8	70279851	AA	AA	1
1303	CH8MM54	8	70414822	BB	BB	1
1132	CH8MM56	8	71916854	BB	BB	1
593	CH8MM57	8	73705878	BB	BB	1
1565	CH8MM58	8	75853635	AA	AA	1
1618	CH8MM60	8	76020113	AA	AA	1
1281	CH8MM59	8	76424930	BB	BB	1
1292	CH8MM61	8	78751914	AA	AA	1
138	CH8MM62	8	79895387	BB	BB	1
1799	CH8MM63	8	80710527	AA	AA	1
1566	CH8MM64	8	81712094	AA	AA	1
973	CH8MM65	8	82781758	AA	AA	1
594	CH8MM66	8	83666392	BB	BB	1
595	CH8MM67	8	85792899	BB	BB	1
1759	CH8MM68	8	86274191	AA	AA	1
596	CH8MM69	8	87473707	AA	AA	1
597	CH8MM70	8	87829870	BB	BB	1
598	CH8MM71	8	88385924	BB	BB	1
1897	CH8MM72	8	90580555	AA	AA	1
1039	CH8MM73BLK12	8	90954211	AA	AA	1
599	CH8MM74	8	93256446	BB	BB	1
1489	CH8MM75	8	95416227	AA	AA	1
1785	CH8MM76	8	96704003	AA	AA	1
1096	CH8MM77	8	97015295	BB	BB	1
1787	CH8MM78	8	98119407	AA	AA	1
600	CH8MM79	8	100032524	AA	AA	1
1340	CH8MM80	8	102146578	BB	BB	1
1135	CH8MM81	8	102776851	AA	AA	1
601	CH8MM82	8	103059131	BB	BB	1
1335	CH8MM83	8	104062947	AA	AA	1
177	CH8MM84	8	107533038	AA	AA	1
178	CH8MM85	8	109495249	BB	BB	1
1101	CH8MM86	8	110138905	AA	AA	1
1733	CH8MM87	8	110744101	AA	AA	1
602	CH8MM88	8	114439370	AA	AA	1
1567	CH8MM89	8	115100673	BB	BB	1
179	CH8MM90	8	117534645	AA	AA	1
987	CH8MM91	8	117633985	BB	BB	1
603	CH8MM92	8	119309883	BB	BB	1
1358	CH8MM93	8	122486972	AA	AA	1
1875	CH8MM94	8	123077700	BB	BB	1
1338	CH8MM95	8	124695307	AA	AA	1
1608	CH8MM96	8	126222738	BB	BB	1
1909	CH8MM97	8	127309165	AA	AA	1
1290	CH8MM98	8	128556740	AA	AA	1
1044	CH9MM1BLK1	9	6238771	AA	AA	1
239	CH9MM2	9	6854122	BB	BB	1
937	CH9MM3	9	8898196	BB	BB	1
604	CH9MM4	9	9290514	BB	BB	1
268	CH9MM5	9	10573036	AA	AA	1
259	CH9MM6	9	12359728	BB	BB	1
285	CH9MM8	9	13934381	AA	AA	1

180	CH9MM9	9	17937120	BB	BB	1
605	CH9MM10	9	19033659	AA	AA	1
1972	CH9MM11	9	21871858	BB	BB	1
606	CH9MM12	9	23319649	BB	BB	1
607	CH9MM13	9	24700666	AA	AA	1
1017	CH9MM14	9	24909258	AA	AA	1
608	CH9MM15	9	26517644	BB	BB	1
1231	CH9MM16	9	26736735	AA	AA	1
1919	CH9MM17	9	27976698	NC	NC	NA
1885	CH9MM18	9	29551530	AA	AA	1
1642	CH9MM19	9	29739642	BB	BB	1
103	CH9MM20	9	29852258	AA	AA	1
938	CH9MM21	9	31018400	AA	AA	1
3	CH9MM22GM2	9	32666424	AA	AA	1
1122	CH9MM23	9	32749628	BB	BB	1
284	CH9MM24	9	33392733	BB	BB	1
1059	CH9MM25	9	33590882	AA	AA	1
1386	CH9MM26	9	34370090	AA	AA	1
1999	CH9MM27	9	35123703	AA	AA	1
609	CH9MM28	9	36858360	AA	AA	1
1702	CH9MM29	9	36980052	AA	AA	1
1929	CH9MM30	9	37852690	BB	BB	1
610	CH9MM31	9	39635608	BB	BB	1
1404	CH9MM32	9	40618361	BB	BB	1
309	CH9MM34	9	44548116	AA	AA	1
1645	CH9MM35	9	44819837	BB	BB	1
1980	CH9MM36	9	45095016	BB	BB	1
611	CH9MM37	9	46439871	AA	AA	1
1903	CH9MM38	9	47326735	AA	AA	1
181	CH9MM39	9	48120628	AA	AA	1
104	CH9MM40	9	48978549	AA	AA	1
1370	CH9MM41	9	49175067	AA	AA	1
612	CH9MM42	9	50092390	BB	BB	1
1458	CH9MM43	9	52426099	AA	AA	1
1126	CH9MM45	9	56494245	BB	BB	1
613	CH9MM46	9	57726154	BB	BB	1
1721	CH9MM47	9	59894599	AA	AA	1
1852	CH9MM48GM3	9	60412482	AA	AA	1
1401	CH9MM49	9	61063765	BB	BB	1
1057	CH9MM51	9	65372396	AA	AA	1
1670	CH9MM52	9	68045771	BB	BB	1
1446	CH9MM53	9	70939731	BB	BB	1
1260	CH9MM54	9	71559961	AA	AA	1
614	CH9MM55	9	72736142	BB	BB	1
1469	CH9MM56	9	74795303	BB	BB	1
615	CH9MM57	9	76480826	BB	BB	1
1956	CH9MM58	9	78240837	AA	AA	1
1758	CH9MM60	9	78570158	AA	AA	1
999	CH9MM59BLK9	9	78806397	BB	BB	1
182	CH9MM61	9	79962408	BB	BB	1
1720	CH9MM62	9	81662486	BB	BB	1
1173	CH9MM63	9	83002386	BB	BB	1
616	CH9MM64	9	84006171	AA	AA	1
1267	CH9MM65	9	85037758	BB	BB	1
1312	CH9MM66	9	86187152	AA	AA	1
617	CH9MM67	9	87303960	AA	AA	1
618	CH9MM68	9	87742958	BB	BB	1
1139	CH9MM69	9	87767598	AA	AA	1
1779	CH9MM70	9	88089695	BB	BB	1
183	CH9MM71	9	90565254	BB	BB	1
619	CH9MM72	9	92109531	AA	AA	1
1796	CH9MM73GM4	9	92970608	AA	AA	1
620	CH9MM74	9	94166392	BB	BB	1
105	CH9MM75	9	95908047	BB	BB	1
1244	CH9MM76	9	96294531	AA	AA	1
621	CH9MM77	9	98461315	BB	BB	1
1886	CH9MM78	9	99747904	AA	AA	1
1420	CH9MM79	9	101861993	AA	AA	1
622	CH9MM81	9	103502618	AA	AA	1
1380	CH9MM82	9	105368812	AA	AA	1
1623	CH9MM83	9	105862911	AA	AA	1
623	CH9MM84	9	106162074	BB	BB	1
919	CH9MM85	9	107973150	BB	BB	1
624	CH9MM86	9	108560373	AA	AA	1
625	CH9MM87	9	109706053	AA	AA	1
626	CH9MM88	9	111687776	BB	BB	1
184	CH9MM89	9	113120186	BB	BB	1
1266	CH9MM90	9	113854986	AA	AA	1
1652	CH9MM91	9	113918336	BB	BB	1

1821	CH9MM92	9	114985043	AA	AA	1
1138	CH9MM93	9	117747782	BB	BB	1
294	CH9MM94	9	121744927	AA	AA	1
1804	CH9MM95	9	122983055	BB	BB	1
4	CH10MM5GM1	10	3758624	BB	BB	1
1676	CH10MM4	10	4917918	BB	BB	1
627	CH10MM3	10	5527504	AA	AA	1
32	CH10MM2	10	6309157	AA	AA	1
1954	CH10MM6	10	6738688	BB	BB	1
1447	CH10MM1	10	6953955	BB	BB	1
1114	CH10MM7	10	9484968	AA	AA	1
1225	CH10MM8	10	10700784	AA	AA	1
1568	CH10MM9	10	12884895	BB	BB	1
1569	CH10MM10	10	14278380	AA	AA	1
1230	CH10MM11GM2	10	14279080	AA	AA	1
1304	CH10MM12	10	15676314	BB	BB	1
1390	CH10MM13	10	17819300	AA	AA	1
1278	CH10MM14	10	18064006	AA	AA	1
1599	CH10MM15	10	18688500	BB	BB	1
628	CH10MM16	10	19492053	BB	BB	1
295	CH10MM17	10	20201379	AA	AA	1
31	CH10MM18	10	21127405	BB	BB	1
1195	CH10MM19	10	22797128	AA	AA	1
629	CH10MM20	10	24363410	AA	AA	1
995	CH10MM21BLK4	10	25086456	AA	AA	1
1347	CH10MM22GM3	10	25412501	BB	BB	1
630	CH10MM23	10	25826735	BB	BB	1
296	CH10MM24	10	26673653	BB	BB	1
631	CH10MM25	10	28011155	AA	AA	1
1898	CH10MM26	10	28325761	BB	BB	1
185	CH10MM27	10	28833665	AA	AA	1
632	CH10MM29GM4	10	30882088	AA	AA	1
633	CH10MM30	10	34453799	BB	BB	1
634	CH10MM31	10	36073986	AA	AA	1
635	CH10MM32	10	38841497	BB	BB	1
1416	CH10MM33	10	42577994	AA	AA	1
636	CH10MM34	10	44182365	BB	BB	1
33	CH10MM35	10	46576862	AA	AA	1
1179	CH10MM36	10	46828627	BB	BB	1
976	CH10MM37	10	48092341	BB	BB	1
1881	CH10MM38	10	48561820	AA	AA	1
186	CH10MM39	10	50845564	AA	AA	1
1279	CH10MM40	10	53619196	AA	AA	1
1681	CH10MM41	10	55664571	AA	AA	1
1650	CH10MM42	10	57441761	BB	BB	1
1870	CH10MM43	10	60941049	BB	BB	1
1854	CH10MM44	10	64390724	AA	AA	1
1455	CH10MM45	10	66279868	BB	BB	1
637	CH10MM46	10	67656873	BB	BB	1
1210	CH10MM47	10	68025724	BB	BB	1
638	CH10MM48	10	69280498	AA	AA	1
1207	CH10MM49	10	69881567	AA	AA	1
988	CH10MM50	10	71019591	BB	BB	1
34	CH10MM51	10	71793759	BB	AB	0.5
639	CH10MM52	10	74448258	BB	AB	0.5
640	CH10MM53	10	76548630	AA	AA	1
641	CH10MM55	10	80485287	BB	BB	1
14	CH10MM54BLK11	10	80631150	AA	AA	1
1419	CH10MM56	10	82828700	AA	AA	1
642	CH10MM57	10	84521195	BB	BB	1
1199	CH10MM58	10	85178261	BB	BB	1
1958	CH10MM60	10	86136706	BB	BB	1
35	CH10MM61	10	86423967	BB	BB	1
1021	CH10MM62	10	89516856	BB	BB	1
970	CH10MM63	10	89974158	BB	BB	1
643	CH10MM64	10	90896067	BB	BB	1
187	CH10MM65	10	92809785	AA	AA	1
644	CH10MM66	10	93352098	BB	BB	1
1959	CH10MM67	10	93982622	BB	BB	1
645	CH10MM68	10	94352518	AA	AA	1
646	CH10MM69	10	97131553	BB	BB	1
647	CH10MM70	10	97485416	AA	AA	1
1628	CH10MM71	10	98377524	AA	AA	1
1671	CH10MM72	10	98695109	AA	AA	1
1216	CH10MM73	10	99116064	BB	BB	1
1692	CH10MM74	10	99409366	AA	AA	1
1373	CH10MM75	10	99950661	AA	AA	1
648	CH10MM76	10	102211202	AA	AA	1
649	CH10MM77	10	103889063	BB	BB	1

1236	CH10MM78	10	104351918	BB	BB	1
650	CH10MM79	10	105836407	AA	AA	1
1050	CH10MM80	10	107039892	BB	BB	1
1792	CH10MM81	10	107826818	AA	AA	1
1743	CH10MM82	10	108573655	AA	AA	1
1202	CH10MM83	10	114241265	BB	BB	1
651	CH10MM84	10	114578015	BB	BB	1
1699	CH10MM85	10	114709780	BB	BB	1
652	CH10MM86	10	115637466	AA	AA	1
1757	CH10MM87	10	117387550	BB	BB	1
256	CH10MM88	10	117648211	AA	AA	1
271	CH10MM89	10	119162331	BB	BB	1
30	CH10MM90	10	119750707	AA	AA	1
653	CH10MM91	10	122967275	AA	AA	1
232	CH10MM92	10	126134642	BB	BB	1
1289	CH10MM93	10	126648831	AA	AA	1
1176	CH10MM94	10	128045868	BB	BB	1
654	CH11MM1	11	3504147	BB	BB	1
655	CH11MM2	11	3836944	AA	AA	1
1209	CH11MM3	11	4630282	BB	BB	1
1682	CH11MM5	11	6362207	AA	AA	1
656	CH11MM6	11	7573952	AA	AA	1
657	CH11MM7	11	9243618	BB	BB	1
1006	CH11MM8	11	11121093	AA	AA	1
1110	CH11MM9	11	12243247	AA	AA	1
658	CH11MM10	11	12245757	AA	AA	1
1596	CH11MM11	11	13463046	BB	BB	1
36	CH11MM12	11	15047305	AA	AA	1
1810	CH11MM13	11	15702375	AA	AA	1
15	CH11MM14	11	17549493	AA	AA	1
1417	CH11MM15	11	18054381	BB	BB	1
188	CH11MM16	11	18062221	AA	AA	1
1461	CH11MM17	11	19418926	AA	AA	1
1937	CH11MM18	11	20271242	BB	BB	1
1007	CH11MM19	11	20903251	AA	AA	1
659	CH11MM20	11	22764065	BB	BB	1
660	CH11MM21	11	25068970	AA	AA	1
1462	CH11MM22	11	26572348	AA	AA	1
1346	CH11MM23	11	28364810	AA	AA	1
1315	CH11MM24	11	30321273	BB	BB	1
297	CH11MM25	11	31008902	AA	AA	1
1184	CH11MM26	11	32377700	BB	BB	1
1460	CH11MM27	11	33001267	AA	AA	1
1740	CH11MM28	11	34910669	AA	AA	1
1625	CH11MM29BLK3	11	35311958	AA	AA	1
661	CH11MM30	11	36344694	AA	AA	1
662	CH11MM31	11	37755798	BB	BB	1
1853	CH11MM32	11	38442942	AA	AA	1
1663	CH11MM33	11	40194968	AA	AA	1
1818	CH11MM34GM1	11	40317937	AA	AA	1
1586	CH11MM36	11	41102890	BB	BB	1
1323	CH11MM35	11	41218506	AA	AA	1
1049	CH11MM37	11	44098948	AA	AA	1
663	CH11MM38	11	46419243	BB	BB	1
664	CH11MM39	11	46819291	BB	BB	1
1697	CH11MM40	11	50636044	AA	AA	1
898	CH11MM41GM2	11	53500413	AA	AA	1
908	CH11MM42	11	53913898	BB	BB	1
665	CH11MM43	11	54612623	BB	BB	1
666	CH11MM44	11	54789660	AA	AA	1
1570	CH11MM45	11	55285381	AA	AA	1
1219	CH11MM46	11	56301734	BB	BB	1
1018	CH11MM47	11	56406894	AA	AA	1
667	CH11MM48	11	58135145	BB	BB	1
1291	CH11MM49	11	58458377	BB	BB	1
1591	CH11MM50	11	58989824	BB	BB	1
1381	CH11MM51	11	61343391	AA	AA	1
668	CH11MM52	11	62883393	BB	BB	1
669	CH11MM53	11	65091595	AA	AA	1
670	CH11MM54	11	66609628	AA	AA	1
671	CH11MM55	11	68661293	BB	BB	1
672	CH11MM56	11	69711205	BB	BB	1
1695	CH11MM57	11	70068157	BB	BB	1
1264	CH11MM58GM3	11	70597539	AA	AA	1
673	CH11MM59	11	72725194	BB	BB	1
1320	CH11MM60	11	73154451	AA	AA	1
674	CH11MM61	11	74001043	BB	BB	1
675	CH11MM62	11	76485930	BB	BB	1
940	CH11MM63	11	76675746	AA	AA	1

941	CH11MM64	11	76688539	BB	BB	1
939	CH11MM65	11	76700144	BB	BB	1
140	CH11MM66GM4-BM	11	78262029	BB	BB	1
16	CH11MM67BLK10	11	79143057	BB	BB	1
676	CH11MM68	11	79438025	BB	BB	1
1762	CH11MM69	11	82498763	AA	AA	1
677	CH11MM70	11	83303305	AA	AA	1
1092	CH11MM71	11	84623195	AA	AA	1
1055	CH11MM72	11	86651953	BB	BB	1
1438	CH11MM73	11	86849708	BB	BB	1
678	CH11MM74	11	88326517	BB	BB	1
1240	CH11MM75	11	90427362	AA	AA	1
679	CH11MM76	11	92352034	BB	BB	1
1019	CH11MM77	11	93055788	BB	BB	1
680	CH11MM78	11	95518878	AA	AA	1
1371	CH11MM79	11	96416258	AA	AA	1
681	CH11MM80	11	96495692	AA	AA	1
189	CH11MM81	11	98372304	AA	AA	1
1089	CH11MM82	11	98810268	AA	AA	1
1023	CH11MM83	11	99941656	AA	AA	1
1901	CH11MM84	11	103491945	AA	AA	1
1294	CH11MM85	11	105128129	AA	AA	1
1888	CH11MM86	11	106869201	AA	AA	1
1864	CH11MM87	11	109285573	BB	BB	1
682	CH11MM88	11	110339907	BB	AA	0
683	CH11MM89	11	111051990	AA	BB	0
1156	CH11MM90	11	112125029	AA	BB	0
1297	CH11MM91	11	113894515	AA	AA	1
684	CH11MM92	11	115791803	AA	AA	1
1393	CH11MM93	11	120958376	BB	BB	1
5	CH12MM1GM1	12	3596756	AA	AA	1
1397	CH12MM2	12	4885108	BB	BB	1
1047	CH12MM3	12	5294765	BB	BB	1
685	CH12MM4	12	5765767	AA	AA	1
1190	CH12MM5	12	7463965	BB	BB	1
1366	CH12MM6	12	9700479	AA	AA	1
1797	CH12MM7	12	9998201	AA	AA	1
686	CH12MM8	12	10650268	AA	AA	1
687	CH12MM9	12	11965329	AA	AA	1
1429	CH12MM10	12	12044042	BB	BB	1
1051	CH12MM11	12	13433079	BB	BB	1
688	CH12MM12	12	14919260	BB	BB	1
1342	CH12MM13	12	16632291	AA	AA	1
689	CH12MM14	12	16730360	BB	BB	1
690	CH12MM15	12	17624546	BB	BB	1
1421	CH12MM16	12	24778980	BB	BB	1
1827	CH12MM22	12	26183320	AA	AA	1
978	CH12MM23	12	26211845	BB	BB	1
691	CH12MM17	12	28030668	BB	BB	1
1022	CH12MM18	12	29050535	AA	AA	1
1988	CH12MM19	12	29970571	BB	BB	1
692	CH12MM20	12	30248600	BB	BB	1
1722	CH12MM21	12	30933537	BB	BB	1
990	CH12MM27	12	31581541	AA	AA	1
1718	CH12MM24	12	34232745	BB	BB	1
1383	CH12MM25	12	35470170	BB	BB	1
693	CH12MM26	12	35676699	BB	BB	1
1808	CH12MM28	12	37060797	AA	AA	1
1068	CH12MM29	12	38140061	AA	AA	1
972	CH12MM34BLK4	12	38917909	AA	AA	1
694	CH12MM30	12	39880986	AA	AA	1
695	CH12MM31	12	39984728	AA	AA	1
696	CH12MM32	12	40506398	AA	AA	1
38	CH12MM33	12	43760942	AA	AA	1
1477	CH12MM35	12	44401447	BB	BB	1
39	CH12MM36	12	45531038	BB	BB	1
190	CH12MM37	12	47974230	BB	BB	1
697	CH12MM38	12	50389232	AA	AA	1
698	CH12MM39	12	52566442	AA	AA	1
1989	CH12MM40	12	52606834	BB	BB	1
1144	CH12MM41	12	53006058	BB	BB	1
1317	CH12MM42	12	53966632	BB	BB	1
699	CH12MM43	12	56053114	AA	AA	1
1341	CH12MM44	12	57999477	BB	BB	1
229	CH12MM45	12	58987423	BB	BB	1
700	CH12MM46	12	61887457	AA	AA	1
191	CH12MM47	12	63249337	BB	BB	1
1185	CH12MM48	12	64643832	AA	AA	1
701	CH12MM49	12	67263101	BB	BB	1

1838	CH12MM50	12	67805946	AA	AA	1
1349	CH12MM51	12	68839023	AA	AA	1
1883	CH12MM57GM2	12	69207595	BB	BB	1
702	CH12MM52	12	69650523	BB	BB	1
1693	CH12MM53	12	70281043	AA	AA	1
703	CH12MM54	12	71507618	AA	AA	1
704	CH12MM55	12	72933972	BB	BB	1
1056	CH12MM56	12	74772125	AA	AA	1
705	CH12MM58	12	76425087	AA	AA	1
192	CH12MM59	12	78650250	BB	BB	1
1100	CH12MM60	12	79308253	BB	BB	1
706	CH12MM61	12	80709328	BB	BB	1
707	CH12MM62	12	82213253	AA	AA	1
708	CH12MM63	12	83823782	AA	AA	1
288	CH12MM65	12	84362672	BB	BB	1
17	CH12MM68BLK10	12	85692061	BB	BB	1
1414	CH12MM64	12	87092310	AA	AA	1
1626	CH12MM71	12	89968959	AA	AA	1
40	CH12MM66	12	90125552	AA	AA	1
1141	CH12MM67	12	91305812	AA	AA	1
709	CH12MM69	12	91935453	BB	BB	1
1703	CH12MM70	12	94329626	AA	AA	1
710	CH12MM72	12	96071736	AA	AA	1
711	CH12MM73	12	98045373	AA	AA	1
712	CH12MM74	12	99546237	BB	BB	1
1412	CH12MM75	12	99830551	AA	AA	1
713	CH12MM76	12	101113713	BB	BB	1
1309	CH12MM77	12	101725877	BB	BB	1
714	CH12MM78	12	102494711	AA	AA	1
1973	CH12MM82	12	104206840	BB	BB	1
1974	CH12MM79	12	104360190	BB	BB	1
715	CH12MM80	12	106362513	AA	AA	1
1896	CH12MM81	12	108645309	AA	AA	1
37	CH12MM83	12	109763283	AA	AA	1
716	CH12MM84	12	112079425	BB	BB	1
717	CH12MM85	12	112469664	BB	BB	1
994	CH12MM86GM3	12	113892272	BB	BB	1
1013	CH12MM87GM4	12	116142864	BB	BB	1
1255	CH12MM88	12	117682799	AA	AA	1
1830	CH12MM89	12	119155237	AA	AA	1
257	CH5MM1	13	3183487	AA	BB	0
1712	CH13MM1	13	3834369	BB	AA	0
718	CH13MM2	13	4519481	BB	AA	0
719	CH13MM3GM1	13	5448989	BB	AA	0
720	CH13MM4	13	5635648	BB	AA	0
721	CH13MM5BLK1	13	6354041	AA	BB	0
1802	CH13MM6	13	7753665	BB	BB	1
722	CH13MM7	13	9436457	BB	BB	1
723	CH13MM8	13	10877002	AA	AA	1
724	CH13MM9	13	13809787	BB	BB	1
1840	CH13MM10	13	15585691	BB	BB	1
725	CH13MM11	13	16515352	BB	BB	1
1192	CH13MM13	13	17463623	BB	BB	1
1448	CH13MM14	13	17552109	BB	BB	1
726	CH13MM15	13	19323344	AA	AA	1
1812	CH13MM16	13	20305361	AA	AA	1
1198	CH13MM17	13	21090638	BB	BB	1
1967	CH13MM18	13	21472397	AA	AA	1
1106	CH13MM19	13	23269313	AA	AA	1
1968	CH13MM20	13	23889476	BB	BB	1
1775	CH13MM21	13	26108984	AA	AA	1
41	CH13MM22	13	27865697	BB	BB	1
727	CH13MM23	13	30861497	BB	BB	1
1374	CH13MM24	13	31457302	AA	AA	1
1382	CH13MM25	13	32178311	AA	AA	1
929	CH13MM26	13	33045669	AA	AA	1
1351	CH13MM27	13	34364143	BB	BB	1
728	CH13MM28GM2	13	34398760	AA	AA	1
1470	CH13MM29	13	35246746	BB	BB	1
729	CH13MM30	13	36741591	AA	AA	1
1760	CH13MM31	13	37431044	AA	AA	1
193	CH13MM32	13	37703036	BB	BB	1
194	CH13MM33	13	40192199	BB	BB	1
975	CH13MM34	13	41647851	AA	AA	1
730	CH13MM35	13	42993573	BB	BB	1
22	CH13MM37GM3	13	43184888	AA	AA	1
1394	CH13MM36	13	43324437	AA	AA	1
1235	CH13MM38	13	45512714	AA	AA	1
1926	CH13MM39	13	47283397	BB	BB	1

1744	CH13MM40	13	47969418	AA	AA	1
731	CH13MM41	13	51829505	BB	BB	1
1262	CH13MM42	13	55100592	BB	BB	1
1442	CH13MM43	13	56377339	BB	BB	1
1314	CH13MM44	13	57400953	AA	AA	1
195	CH13MM45	13	58872351	AA	AA	1
196	CH13MM46	13	59592605	AA	AA	1
732	CH13MM47	13	60565406	BB	BB	1
1248	CH13MM48	13	60865804	AA	AA	1
1388	CH13MM49	13	65333750	AA	AA	1
1571	CH13MM50	13	67028550	BB	BB	1
1426	CH13MM51	13	69477270	BB	BB	1
733	CH13MM52	13	71441788	AA	AA	1
734	CH13MM53	13	74508925	AA	AA	1
1969	CH13MM56	13	75280501	AA	AA	1
735	CH13MM54	13	75650435	AA	AA	1
736	CH13MM55	13	77710196	BB	BB	1
197	CH13MM57	13	80553312	AA	AA	1
737	CH13MM58	13	82210489	BB	BB	1
260	CH13MM59	13	84849641	BB	BB	1
1746	CH13MM60	13	85707679	BB	BB	1
1228	CH13MM61	13	86671214	BB	BB	1
198	CH13MM62	13	88285944	BB	BB	1
1052	CH13MM63	13	89731657	BB	BB	1
1867	CH13MM64	13	90806561	BB	BB	1
1791	CH13MM65	13	91283959	AA	AA	1
199	CH13MM66	13	92020130	BB	BB	1
1813	CH13MM67	13	92245473	BB	BB	1
200	CH13MM68	13	92860326	BB	BB	1
738	CH13MM69GM4	13	93139331	BB	BB	1
1726	CH13MM71GM5	13	94895370	BB	BB	1
739	CH13MM70	13	97406695	BB	BB	1
740	CH13MM72	13	98994748	BB	BB	1
1572	CH13MM73	13	100632960	AA	AA	1
741	CH13MM74	13	103558916	AA	AA	1
1894	CH13MM75	13	103971133	BB	BB	1
742	CH13MM76	13	105820240	AA	AA	1
743	CH13MM78	13	108957755	BB	BB	1
996	CH13MM80BLK13	13	109116066	AA	AB	0.5
744	CH13MM79	13	110779023	AA	AA	1
745	CH13MM81	13	112904897	AA	AA	1
201	CH13MM82	13	114048946	AA	AA	1
1749	CH13MM83	13	115303528	AA	AA	1
1814	CH13MM84	13	116065800	BB	BB	1
746	CH13MM85	13	116386648	AA	AA	1
747	CH13MM86	13	118224650	BB	BB	1
279	13MM87BLK14GM6-	13	119511987	BB	BB	0.5
280	13MM88BLK15GM7-	13	120164565	NC	BB	0.5
1280	CH14MM11	14	5106656	AA	AA	1
1439	CH14MM9	14	6241952	BB	BB	1
1794	CH14MM8	14	9923746	AA	AA	1
749	CH14MM7	14	10228293	BB	BB	1
1155	CH14MM5	14	11563922	AA	AA	1
1028	CH14MM4	14	11675467	BB	BB	1
1823	CH14MM3	14	12650357	AA	AA	1
748	CH14MM2GM1	14	13453342	AA	AA	1
1640	CH14MM6	14	14034640	AA	AA	1
1573	CH14MM1	14	14378215	AA	AA	1
1233	CH14MM10	14	19739287	AA	AA	1
750	CH14MM12	14	22770669	BB	BB	1
1254	CH14MM13	14	23860869	AA	AA	1
202	CH14MM14	14	25263173	AA	AA	1
751	CH14MM16	14	26065225	AA	AA	1
1905	CH14MM17	14	27722870	BB	BB	1
752	CH14MM18	14	29200094	BB	BB	1
1574	CH14MM19	14	30905377	AA	AA	1
1634	CH14MM24	14	31206380	BB	BB	1
753	CH14MM20	14	31388998	AA	AA	1
1811	CH14MM21	14	31988147	AA	AA	1
1270	CH14MM22	14	33517979	BB	BB	1
754	CH14MM23	14	35109515	AA	AA	1
1324	CH14MM25	14	37073870	BB	BB	1
755	CH14MM26	14	38870370	AA	AA	1
756	CH14MM27	14	39287167	BB	BB	1
757	CH14MM28	14	40908622	BB	BB	1
758	CH14MM29	14	45938711	BB	BB	1
1930	CH14MM34	14	46629433	BB	BB	1
1638	CH14MM35	14	46850451	AA	AA	1
203	CH14MM30	14	47084921	AA	AA	1

759	CH14MM31	14	48765032	AA	AA	1
261	CH14MM32	14	50465874	BB	BB	1
1127	CH14MM33	14	52378037	BB	BB	1
760	CH14MM36	14	54883754	BB	BB	1
1590	CH14MM42	14	55030573	AA	AA	1
761	CH14MM37	14	55860099	BB	BB	1
762	CH14MM38	14	56064526	AA	AA	1
1258	CH14MM39	14	59052744	AA	AA	1
204	CH14MM40	14	61072511	AA	AA	1
763	CH14MM41	14	62128894	BB	BB	1
1327	CH14MM43	14	63162049	BB	BB	1
1272	CH14MM51	14	65818201	BB	BB	1
764	CH14MM44	14	67215800	BB	BB	1
1154	CH14MM45	14	68583578	AA	AA	1
765	CH14MM46	14	70005182	BB	BB	1
1575	CH14MM47GM2	14	70841558	AA	AA	1
1456	CH14MM48	14	71503241	AA	AA	1
766	CH14MM49	14	72134472	AA	AA	1
1636	CH14MM57BLK4	14	72645830	BB	BB	1
1321	CH14MM50	14	73434912	AA	AA	1
1654	CH14MM52	14	74946682	AA	AA	1
925	CH14MM61	14	75501470	BB	BB	1
298	CH14MM53	14	76155063	BB	BB	1
767	CH14MM54	14	76654459	AA	AA	1
768	CH14MM55	14	79257972	BB	BB	1
1008	CH14MM56	14	80004828	AA	AA	1
42	CH14MM58	14	81618169	BB	BB	1
769	CH14MM59	14	82546798	BB	BB	1
1819	CH14MM60	14	83712290	AA	AA	1
770	CH14MM62	14	86453528	BB	BB	1
1364	CH14MM63	14	87076283	BB	BB	1
771	CH14MM64	14	89153678	BB	BB	1
1904	CH14MM65	14	91308015	BB	BB	1
1795	CH14MM66	14	92248728	BB	BB	1
772	CH14MM67	14	93361881	AA	AA	1
773	CH14MM68	14	97438564	BB	BB	1
1256	CH14MM69	14	100013505	BB	BB	1
1684	CH14MM70	14	100445071	BB	BB	1
1589	CH14MM77BLK8	14	102606376	AA	AA	1
43	CH14MM71	14	102852436	AA	AA	1
1576	CH14MM79GM3	14	104715167	BB	BB	1
774	CH14MM72	14	106124982	AA	AA	1
1048	CH14MM73	14	106509691	AA	AA	1
1189	CH14MM74	14	106646787	AA	AA	1
775	CH14MM75	14	107011190	BB	BB	1
205	CH14MM76	14	107731485	BB	BB	1
1365	CH14MM78	14	111005855	AA	AA	1
776	CH14MM80	14	111918001	AA	AA	1
1476	CH14MM81	14	112181307	BB	BB	1
1755	CH14MM82	14	114027387	BB	BB	1
1666	CH14MM83	14	117495486	AA	AA	1
777	CH14MM84	14	118816308	BB	BB	1
778	CH14MM86	14	120209095	BB	BB	1
1295	CH14MM87	14	121897026	BB	BB	1
779	CH14MM88	14	122290002	AA	AA	1
303	CH15MM1GM1	15	3308613	BB	BB	1
1632	CH15MM2	15	3374506	AA	AA	1
780	CH15MM3	15	3698596	AA	AA	1
304	CH15MM4	15	4007342	AA	AA	1
48	CH15MM5	15	4428738	BB	BB	1
1124	CH15MM6	15	5950803	BB	BB	1
50	CH15MM7	15	8503764	AA	AA	1
51	CH15MM8	15	9788091	AA	AA	1
781	CH15MM9	15	10134681	AA	AA	1
782	CH15MM10	15	11311551	AA	AA	1
984	CH15MM11BLK2	15	11336419	BB	BB	1
18	CH15MM12	15	11447887	BB	BB	1
1387	CH15MM13	15	13219581	AA	AA	1
206	CH15MM14	15	14390555	BB	BB	1
44	CH15MM15	15	14883401	AA	AA	1
45	CH15MM16	15	15577287	BB	BB	1
46	CH15MM17	15	16164712	AA	AA	1
783	CH15MM18	15	16731240	BB	BB	1
1410	CH15MM19	15	19252065	BB	BB	1
784	CH15MM20	15	20942493	AA	AA	1
785	CH15MM21	15	22519241	BB	BB	1
786	CH15MM22	15	24593753	AA	AA	1
787	CH15MM23	15	25450907	BB	BB	1
1099	CH15MM24	15	26053052	BB	BB	1

788	CH15MM25	15	28321890	AA	AA	1
789	CH15MM26	15	29267380	BB	BB	1
790	CH15MM27	15	30322376	AA	AA	1
1128	CH15MM28	15	30760906	AA	AA	1
791	CH15MM29GM2	15	31441516	AA	AA	1
792	CH15MM30	15	32042265	AA	AA	1
1680	CH15MM31	15	34331329	AA	AA	1
47	CH15MM32	15	36435968	BB	BB	1
1271	CH15MM33	15	38484642	AA	AA	1
793	CH15MM34	15	38987563	BB	BB	1
1211	CH15MM35	15	41251209	BB	BB	1
49	CH15MM36	15	43051575	AA	AA	1
794	CH15MM37	15	43734239	BB	BB	1
1166	CH15MM38	15	44085745	AA	AA	1
795	CH15MM39	15	45506490	AA	AA	1
796	CH15MM40	15	47030006	AA	AA	1
797	CH15MM41	15	49861772	BB	BB	1
1072	CH15MM42	15	51246783	AA	AA	1
1355	CH15MM43	15	52346737	AA	AA	1
798	CH15MM44	15	52885912	BB	BB	1
1612	CH15MM45	15	53361550	BB	BB	1
799	CH15MM46	15	53899146	BB	BB	1
1910	CH15MM48	15	56669829	AA	AA	1
19	CH15MM47BLK7	15	57023883	AA	AA	1
800	CH15MM49	15	58488373	BB	BB	1
1213	CH15MM50	15	61074302	BB	BB	1
801	CH15MM52	15	61300766	BB	BB	1
286	CH15MM51	15	61445247	BB	BB	1
1182	CH15MM53	15	63333414	BB	BB	1
1879	CH15MM54	15	65460101	AA	AA	1
802	CH15MM55	15	67748939	AA	AA	1
803	CH15MM56	15	68052609	AA	AA	1
804	CH15MM57	15	70372976	AA	AA	1
1301	CH15MM58	15	71208332	BB	BB	1
805	CH15MM59	15	71789079	BB	BB	1
806	CH15MM60	15	72083757	BB	BB	1
1961	CH15MM61	15	74721799	BB	BB	1
1083	CH15MM62	15	77708741	BB	BB	1
1318	CH15MM63	15	78235888	AA	AA	1
807	CH15MM64	15	78643361	BB	BB	1
1121	CH15MM65	15	79277370	AA	AA	1
1777	CH15MM66	15	81710876	AA	AA	1
808	CH15MM68	15	82103452	BB	BB	1
1936	CH15MM67	15	82287795	BB	BB	1
1085	CH15MM69	15	84586258	AA	AA	1
1203	CH15MM70GM3	15	85654981	AA	AA	1
809	CH15MM71	15	85829790	BB	BB	1
810	CH15MM72	15	88354983	AA	AA	1
811	CH15MM73	15	90307637	AA	AA	1
1826	CH15MM74	15	90430095	AA	AA	1
812	CH15MM75	15	92143897	AA	AA	1
1221	CH15MM76	15	93655647	AA	AA	1
1431	CH15MM77	15	95149425	AA	AA	1
1783	CH15MM78	15	95212377	AA	AA	1
813	CH15MM79	15	97009352	AA	AA	1
814	CH15MM80	15	99692230	AA	AA	1
815	CH15MM81	15	101458983	BB	BB	1
1009	CH15MM82	15	101719208	BB	BB	1
816	CH15MM83	15	102859966	BB	BB	1
1501	CH16MM1	16	4144474	BB	BB	1
1502	CH16MM2	16	4667972	BB	BB	1
1577	CH16MM3	16	6041534	AA	AA	1
6	CH16MM4GM1	16	6063341	AA	AA	1
1503	CH16MM5	16	7780694	BB	BB	1
1504	CH16MM6	16	9388035	BB	BB	1
1505	CH16MM7	16	10691888	AA	AA	1
1506	CH16MM8	16	12290643	BB	BB	1
1507	CH16MM9	16	13218611	AA	AA	1
276	CH16MM10GM2-BM	16	15656995	BB	BB	1
1935	CH16MM11	16	18230233	AA	AA	1
1508	CH16MM12	16	19701830	AA	AB	0.5
1509	CH16MM13	16	21142393	AA	AB	0.5
1510	CH16MM14	16	23994437	BB	BB	1
1511	CH16MM15	16	24166418	AA	AA	1
1512	CH16MM16	16	25226052	BB	BB	1
1513	CH16MM17GM3	16	26881632	AA	AA	1
1514	CH16MM18	16	27255016	BB	BB	1
231	CH16MM19	16	28671112	BB	BB	1
1619	CH16MM21BLK4	16	32098801	AA	AA	1

1181	CH16MM23GM4	16	32244260	AA	AA	1
1515	CH16MM22	16	32290630	AA	AA	1
1516	CH16MM24	16	34128681	AA	AA	1
1517	CH16MM25	16	35482510	AA	AA	1
1518	CH16MM26GM5	16	36335823	BB	BB	1
270	CH16MM27	16	37173131	BB	BB	1
1519	CH16MM29	16	40503586	BB	BB	1
1520	CH16MM30GM6	16	41923491	AA	AA	1
1521	CH16MM31	16	42154839	BB	BB	1
1522	CH16MM32	16	45112257	BB	BB	1
1523	CH16MM33	16	45873021	BB	BB	1
1524	CH16MM34	16	47017812	AA	AA	1
1525	CH16MM35	16	50118929	AA	AA	1
1615	CH16MM36	16	50190187	BB	BB	1
1526	CH16MM37	16	50548211	BB	BB	1
1527	CH16MM38GM7	16	50871462	BB	BB	1
230	CH16MM39	16	51262496	AA	AA	1
1282	CH16MM40	16	51497066	AA	AA	1
1662	CH16MM41	16	53261569	BB	BB	1
136	CH16MM42	16	54913621	AA	AA	1
1528	CH16MM43	16	56283272	BB	BB	1
1529	CH16MM44GM8	16	56495501	BB	BB	1
1530	CH16MM45	16	57112848	AA	AA	1
1531	CH16MM47	16	57372473	BB	BB	1
1532	CH16MM46BLK7	16	57549483	AA	AA	1
1533	CH16MM48	16	58953458	BB	BB	1
1534	CH16MM49	16	60798648	BB	BB	1
1535	CH16MM50	16	62618045	AA	AA	1
1635	CH16MM51	16	64722595	BB	BB	1
1536	CH16MM52	16	66093444	AA	AA	1
1537	CH16MM53	16	67060136	AA	AA	1
1538	CH16MM54	16	68808348	AA	AA	1
1539	CH16MM55	16	70491734	BB	BB	1
1540	CH16MM56	16	72307864	AA	AA	1
1541	CH16MM58	16	73993274	BB	BB	1
1542	CH16MM59	16	76171216	AA	AA	1
1422	CH16MM60	16	76664493	BB	BB	1
1543	CH16MM61	16	77930175	BB	BB	1
1971	CH16MM62	16	78831258	BB	BB	1
1817	CH16MM63	16	79289674	AA	AA	1
1544	CH16MM64	16	80626961	AA	AA	1
1545	CH16MM65	16	82578350	BB	BB	1
1620	CH16MM66	16	83578326	BB	BB	1
1201	CH16MM67	16	84350326	BB	BB	1
1546	CH16MM68	16	84894077	AA	AA	1
1547	CH16MM69	16	86259038	AA	AA	1
1548	CH16MM70	16	86908624	AA	AA	1
1549	CH16MM71	16	87295613	AA	AA	1
281	CH16MM72	16	87954873	BB	BB	1
1550	CH16MM73	16	90095207	BB	BB	1
1551	CH16MM74	16	92796504	AA	AA	1
1361	CH16MM75	16	92992901	BB	BB	1
1389	CH16MM76	16	93334996	BB	BB	1
1287	CH16MM80	16	97196709	AA	AA	1
1978	CH16MM77	16	97377184	AA	AA	1
1979	CH16MM78	16	97392186	AA	AA	1
1977	CH16MM79	16	97413001	AA	AA	1
1485	CH17MM1	17	3439137	BB	BB	1
1738	CH17MM2	17	3817191	AA	AA	1
817	CH17MM3	17	4095011	BB	BB	1
818	CH17MM4	17	4643708	AA	AA	1
1045	CH17MM5	17	4712766	BB	BB	1
1990	CH17MM6	17	6089241	BB	BB	1
1694	CH17MM7	17	9189356	AA	AA	1
1098	CH17MM8	17	10192511	AA	AA	1
1601	CH17MM9	17	11452179	BB	BB	1
1102	CH17MM10	17	12534110	BB	BB	1
299	CH17MM11	17	12803351	AA	AA	1
52	CH17MM12	17	14030120	BB	BB	1
1153	CH17MM13	17	14419606	BB	BB	1
1480	CH17MM14	17	16622904	AA	AA	1
819	CH17MM15	17	17844555	BB	BB	1
944	CH17MM16	17	18066517	BB	BB	1
820	CH17MM17	17	21066059	BB	BB	1
1284	CH17MM18	17	22899986	AA	AA	1
1328	CH17MM19	17	24548668	BB	BB	1
1437	CH17MM20	17	25721473	BB	BB	1
300	CH17MM21	17	26432840	BB	BB	1
1907	CH17MM22	17	27670389	BB	BB	1

1884	CH17MM24GM1	17	29272346	AA	AA	1
1261	CH17MM23	17	29999048	BB	BB	1
1161	CH17MM25	17	33329194	BB	BB	1
1010	CH17MM26GM2	17	34415919	AA	AA	1
1951	CH17MM28	17	35013951	BB	BB	1
1208	CH17MM27	17	36145026	BB	BB	1
1800	CH17MM29	17	36858358	AA	AA	1
1932	CH17MM30	17	37300215	BB	BB	1
53	CH17MM31	17	39237947	AA	AA	1
1895	CH17MM32	17	39301872	AA	AA	1
54	CH17MM33	17	41935430	BB	BB	1
1578	CH17MM34	17	43400124	BB	BB	1
1041	CH17MM36BLK2	17	44115026	BB	BB	1
821	CH17MM35	17	44135528	BB	BB	1
822	CH17MM37	17	46051100	BB	BB	1
918	CH17MM40	17	46337718	AA	AA	1
1991	CH17MM38	17	46479965	AA	AA	1
1924	CH17MM39	17	47652414	BB	BB	1
1627	CH17MM43	17	48419593	AA	AA	1
823	CH17MM41	17	48906881	BB	BB	1
824	CH17MM42	17	49864860	AA	AA	1
1311	CH17MM44	17	52918539	AA	AA	1
1772	CH17MM45	17	53789744	BB	BB	1
1402	CH17MM46	17	55577063	AA	AA	1
1408	CH17MM47	17	58978001	BB	BB	1
207	CH17MM48	17	63069234	BB	BB	1
234	CH17MM49	17	63905650	AA	AA	1
1913	CH17MM50	17	64019387	AA	AA	1
301	CH17MM51	17	66168447	BB	BB	1
1483	CH17MM52	17	68041142	BB	BB	1
9	CH17MM55GM3	17	68423635	BB	BB	1
825	CH17MM53	17	68864037	BB	BB	1
1064	CH17MM54	17	69356498	AA	AA	1
1082	CH17MM56	17	70901162	BB	BB	1
1170	CH17MM57	17	71548411	BB	BB	1
1252	CH17MM58	17	72706587	BB	BB	1
287	CH17MM60	17	73410732	BB	BB	1
826	CH17MM59	17	74511779	BB	BB	1
1727	CH17MM61	17	75498192	BB	BB	1
827	CH17MM62	17	75819022	BB	BB	1
1104	CH17MM63	17	77355871	AA	AA	1
1143	CH17MM65	17	79672183	BB	AB	0.5
208	CH17MM66	17	81259640	BB	BB	1
1616	CH17MM67	17	81322892	BB	BB	1
209	CH17MM68	17	82816898	BB	AB	0.5
828	CH17MM69	17	83785490	BB	AB	0.5
829	CH17MM70	17	86193350	BB	AA	0
1579	CH17MM71	17	87117436	AA	BB	0
302	CH17MM72	17	87565416	BB	AA	0
55	CH17MM73	17	88618315	BB	BB	1
830	CH17MM74	17	89195936	AA	AA	1
1649	CH17MM75	17	89506468	BB	BB	1
1790	CH17MM76	17	91886472	AA	BB	0
1906	CH17MM77	17	93502210	AA	AB	0.5
1343	CH17MM78	17	94438440	AA	AA	1
831	CH18MM1	18	3516542	AA	AA	1
832	CH18MM2	18	3796493	BB	BB	1
210	CH18MM3	18	4643091	BB	BB	1
57	CH18MM4	18	5395188	BB	BB	1
1245	CH18MM5	18	8042638	BB	BB	1
1084	CH18MM6	18	9273550	AA	AA	1
211	CH18MM7	18	10209390	AA	AA	1
1580	CH18MM9	18	11084831	AA	AA	1
971	CH18MM8	18	11094900	AA	AA	1
833	CH18MM11	18	12243698	BB	BB	1
1952	CH18MM10	18	12588441	BB	BB	1
248	CH18MM12	18	14663610	AA	AA	1
1858	CH18MM13	18	15141166	AA	AA	1
1851	CH18MM14	18	16547740	AA	AA	1
834	CH18MM15BLK3	18	19422492	AA	AA	1
835	CH18MM17	18	20957267	AA	AA	1
979	CH18MM16	18	21242875	BB	BB	1
1491	CH18MM18	18	22727216	BB	BB	1
212	CH18MM19	18	24575761	BB	BB	1
213	CH18MM20	18	25444458	AA	AA	1
836	CH18MM21	18	26574561	BB	BB	1
837	CH18MM22	18	29090865	AA	AA	1
1215	CH18MM25	18	30637127	BB	BB	1
1592	CH18MM23	18	30765372	AA	AA	1

838	CH18MM24GM1	18	30913598	AA	AA	1
1451	CH18MM26	18	31068735	BB	BB	1
1423	CH18MM27	18	33265241	BB	BB	1
1286	CH18MM28	18	33662244	BB	BB	1
1473	CH18MM29	18	34561612	AA	AA	1
839	CH18MM30	18	36253127	BB	BB	1
1119	CH18MM31	18	37427285	AA	AA	1
1306	CH18MM32	18	39446786	AA	AA	1
840	CH18MM33	18	41041056	AA	AA	1
1174	CH18MM34	18	43056876	BB	AA	0
1581	CH18MM35	18	44155008	BB	AA	0
56	CH18MM36	18	45771525	BB	AA	0
1675	CH18MM37	18	47572175	AA	AA	1
251	CH18MM38	18	49164678	AA	BB	0
1167	CH18MM39	18	51327437	BB	BB	1
214	CH18MM40	18	53236060	BB	AA	0
841	CH18MM41	18	54584928	AA	AA	1
1212	CH18MM42	18	55818301	AA	AA	1
1828	CH18MM43	18	57937237	BB	BB	1
1137	CH18MM44	18	58197988	BB	AA	0
58	CH18MM45	18	59832184	BB	AA	0
1931	CH18MM46	18	62310578	BB	AA	0
1582	CH18MM47	18	62637349	BB	AA	0
1443	CH18MM48	18	63423288	BB	BB	1
1239	CH18MM49	18	64647368	AA	AB	0.5
1820	CH18MM50	18	65300640	AA	AB	0.5
1251	CH18MM51	18	66607940	AA	AB	0.5
1839	CH18MM52	18	68647547	AA	AA	1
1069	CH18MM53	18	68794385	BB	BB	1
842	CH18MM54	18	70256725	BB	BB	1
1661	CH18MM55	18	71583043	AA	AA	1
843	CH18MM56	18	73565434	BB	AB	0.5
1583	CH18MM57	18	74406585	BB	AB	0.5
307	CH18MM58	18	74869249	AA	AB	0.5
1344	CH18MM60	18	77557609	AA	AA	1
1444	CH18MM61	18	79981968	AA	AA	1
7	CH18MM62GM2	18	80818179	AA	AA	1
844	CH18MM63	18	81233507	BB	BB	1
845	CH18MM64	18	82258681	BB	BB	1
20	CH18MM65BLK9	18	84534920	AA	AA	1
263	CH18MM66	18	85538198	AA	AA	1
846	CH18MM67	18	86121066	BB	BB	1
1488	CH18MM68	18	88652070	AA	AA	1
1149	CH19MM1	19	4469681	BB	BB	1
1395	CH19MM2GM1	19	5079820	BB	BB	1
62	CH19MM3	19	5544216	AA	AA	1
1732	CH19MM4	19	6261850	AA	AA	1
21	CH19MM5	19	9004320	BB	BB	1
1333	CH19MM8	19	10041324	BB	BB	1
1016	CH19MM9	19	10043626	BB	BB	1
847	CH19MM6	19	10421408	BB	BB	1
1992	CH19MM7	19	10691690	AA	AA	1
1816	CH19MM11	19	11299589	BB	BB	1
59	CH19MM12	19	13302813	AA	AA	1
848	CH19MM13	19	15443169	BB	BB	1
1229	CH19MM14	19	16858775	AA	AA	1
1719	CH19MM15	19	18502685	BB	BB	1
215	CH19MM16	19	19982142	AA	AA	1
1868	CH19MM17	19	21820516	AA	AA	1
1807	CH19MM18	19	23564161	BB	BB	1
1445	CH19MM19	19	25099261	AA	AA	1
1136	CH19MM21	19	26345166	AA	AA	1
1024	CH19MM22	19	26708877	AA	AA	1
1993	CH19MM23	19	30216574	BB	BB	1
849	CH19MM24GM2	19	30792288	BB	BB	1
983	CH19MM26	19	31461811	AA	AA	1
308	CH19MM25	19	32026360	BB	BB	1
60	CH19MM28	19	33014684	AA	AA	1
61	CH19MM29	19	35297410	BB	BB	1
216	CH19MM30	19	37220834	BB	BB	1
1378	CH19MM31	19	38156894	BB	BB	1
1942	CH19MM32	19	39318915	BB	BB	1
1054	CH19MM33	19	40248051	AA	AA	1
977	CH19MM34BLK4	19	40352976	BB	BB	1
850	CH19MM35	19	41271732	BB	BB	1
1061	CH19MM36	19	42401126	BB	BB	1
1232	CH19MM37	19	43058577	BB	BB	1
851	CH19MM38	19	45300120	BB	BB	1
852	CH19MM39	19	45766920	BB	BB	1

244	CH19MM40	19	46004064	AA	AA	1
853	CH19MM41	19	47361848	AA	AA	1
1011	CH19MM42	19	48126255	BB	BB	1
1688	CH19MM43	19	50117469	BB	BB	1
240	CH19MM44	19	52790214	BB	BB	1
1805	CH19MM45	19	53426500	AA	AA	1
854	CH19MM46	19	55150025	AA	AA	1
1707	CH19MM47	19	56980843	AA	AA	1
1685	CH19MM48	19	59310263	AA	AA	1
1996	CH19MM49	19	60070029	AA	AA	1
8	CH19MM50GM3	19	60746430	BB	BB	1
982	CHXMM1	X	6687680	BB	BB	1
1945	CHXMM3	X	7813261	BB	BB	1
855	CHXMM2	X	9418543	AA	AA	1
130	CHXMM4	X	9869466	AA	AA	1
131	CHXMM5	X	10033359	AA	AA	1
856	CHXMM6	X	10311389	BB	BB	1
133	CHXMM7	X	10566142	BB	BB	1
857	CHXMM9GM1	X	35271739	BB	BB	1
217	CHXMM10	X	37988586	AA	AA	1
218	CHXMM11	X	41313518	AA	AA	1
858	CHXMM12	X	41541725	AA	AA	1
119	CHXMM13	X	46702487	BB	BB	1
859	CHXMM14	X	47251088	AA	AA	1
860	CHXMM17	X	50463583	BB	BB	1
120	CHXMM18	X	51054422	BB	BB	1
1927	CHXMM19	X	51148205	AA	AA	1
861	CHXMM20	X	52039846	BB	BB	1
862	CHXMM21	X	55261255	AA	AA	1
863	CHXMM23	X	56579873	BB	BB	1
219	CHXMM24	X	59866597	BB	BB	1
864	CHXMM25	X	60218954	BB	BB	1
865	CHXMM26	X	61419441	AA	AA	1
989	CHXMM32	X	61560957	BB	BB	1
866	CHXMM27	X	62688042	AA	AA	1
867	CHXMM28	X	63659249	AA	AA	1
121	CHXMM29	X	66992834	AA	AA	1
122	CHXMM30	X	67658652	BB	BB	1
1944	CHXMM39	X	67741556	AA	AA	1
282	CHXMM31	X	68706246	AA	AA	1
1030	CHXMM33	X	70484647	AA	AA	1
868	CHXMM34	X	73251098	BB	BB	1
123	CHXMM35	X	73500498	AA	AA	1
869	CHXMM36	X	74771961	AA	AA	1
124	CHXMM37	X	75467555	AA	AA	1
125	CHXMM38	X	75665787	AA	AA	1
870	CHXMM8	X	77745136	AA	AA	1
220	CHXMM40	X	79762365	BB	BB	1
126	CHXMM41	X	80115206	BB	BB	1
127	CHXMM42	X	80363412	AA	AA	1
871	CHXMM43	X	81244562	AA	AA	1
128	CHXMM45	X	82617765	BB	BB	1
872	CHXMM46	X	83640744	BB	BB	1
873	CHXMM47	X	84362161	AA	AA	1
1474	CHXMM48	X	84542173	AA	AA	1
129	CHXMM49	X	85273099	AA	AA	1
874	CHXMM50	X	85992085	AA	AA	1
221	CHXMM52	X	91719107	AA	AA	1
875	CHXMM53	X	92948478	AA	AA	1
305	CHXMM54	X	93579691	BB	BB	1
222	CHXMM55	X	95226784	BB	BB	1
876	CHXMM56	X	95542610	AA	AA	1
1673	CHXMM57	X	96019952	BB	BB	1
1012	CHXMM67	X	97192077	BB	BB	1
132	CHXMM58	X	97392185	BB	BB	1
877	CHXMM60	X	99463128	BB	BB	1
1982	CHXMM71	X	99685385	BB	BB	1
1983	CHXMM72	X	99723538	AA	AA	1
223	CHXMM61	X	99875599	BB	BB	1
224	CHXMM62	X	101170212	AA	AA	1
878	CHXMM63	X	102703443	BB	BB	1
1970	CHXMM76	X	103344287	BB	BB	1
225	CHXMM64	X	104901192	BB	BB	1
879	CHXMM65	X	105132017	AA	AA	1
880	CHXMM66	X	105253103	AA	AA	1
881	CHXMM68	X	105865711	BB	BB	1
106	CHXMM69	X	107939053	BB	BB	1
107	CHXMM70	X	108069765	BB	BB	1
882	CHXMM73	X	109649518	BB	BB	1

1837	CHXMM75	X	111457480	BB	BB	1
108	CHXMM77	X	114433392	BB	BB	1
1860	CHXMM78	X	117157329	AA	AA	1
883	CHXMM79	X	118714429	AA	AA	1
109	CHXMM80	X	120035863	AA	AA	1
110	CHXMM81	X	120483276	AA	AA	1
993	CHXMM84GM2	X	121847283	BB	BB	1
139	CHXMM82	X	125294088	BB	BB	1
111	CHXMM83	X	126465422	BB	BB	1
1700	CHXMM85	X	129196404	AA	AA	1
1715	CHXMM86	X	129938448	AA	AA	1
884	CHXMM87	X	130181707	AA	AA	1
885	CHXMM88	X	131014211	BB	BB	1
886	CHXMM89	X	132676285	AA	AA	1
1955	CHXMM100	X	133496078	AA	AA	1
1325	CHXMM90	X	133849918	AA	AA	1
112	CHXMM91	X	134629922	BB	BB	1
1288	CHXMM92	X	135117358	BB	BB	1
887	CHXMM93	X	136907718	BB	BB	1
888	CHXMM94	X	138741460	AA	AA	1
889	CHXMM95	X	139579131	BB	BB	1
890	CHXMM96	X	141172394	AA	AA	1
891	CHXMM97	X	141591117	AA	AA	1
113	CHXMM98	X	142416911	BB	BB	1
892	CHXMM99	X	143318561	BB	BB	1
893	CHXMM101	X	145749181	AA	AA	1
894	CHXMM102	X	149491266	AA	AA	1
1610	CHXMM109	X	150151078	BB	BB	1
135	CHXMM22BLK3	X	150253121	AA	AA	1
114	CHXMM103	X	151548019	BB	BB	1
115	CHXMM104	X	152334086	AA	AA	1
116	CHXMM105	X	152491470	AA	AA	1
895	CHXMM106	X	154731164	BB	BB	1
896	CHXMM107	X	156087717	AA	AA	1
1861	CHXMM108	X	158961965	AA	AA	1
117	CHXMM111	X	162861984	AA	AA	1
118	CHXMM112	X	164213115	AA	AA	1
226	CHXMM113	X	165469628	AA	AA	1
227	CHXMM114	X	167610805	BB	BB	1
897	CHXMM115GM3	X	168237748	AA	AA	1
228	CHYMM1BLK1GM1	Y	2107181	BB	NC	NA

	1921
	1996
	96.24%
