# Supplementary figures and images for: Refined cell transfer model reveals roles for Ascl2 and Cxcr3 in splenic localization of mouse NK cells during virus infection
Source: J Immunol. 2025 Jul 22;214(8):1917–25. doi: 10.1093/jimmun/vkaf122 (PMC12313101; doi:10.1093/jimmun/vkaf122)

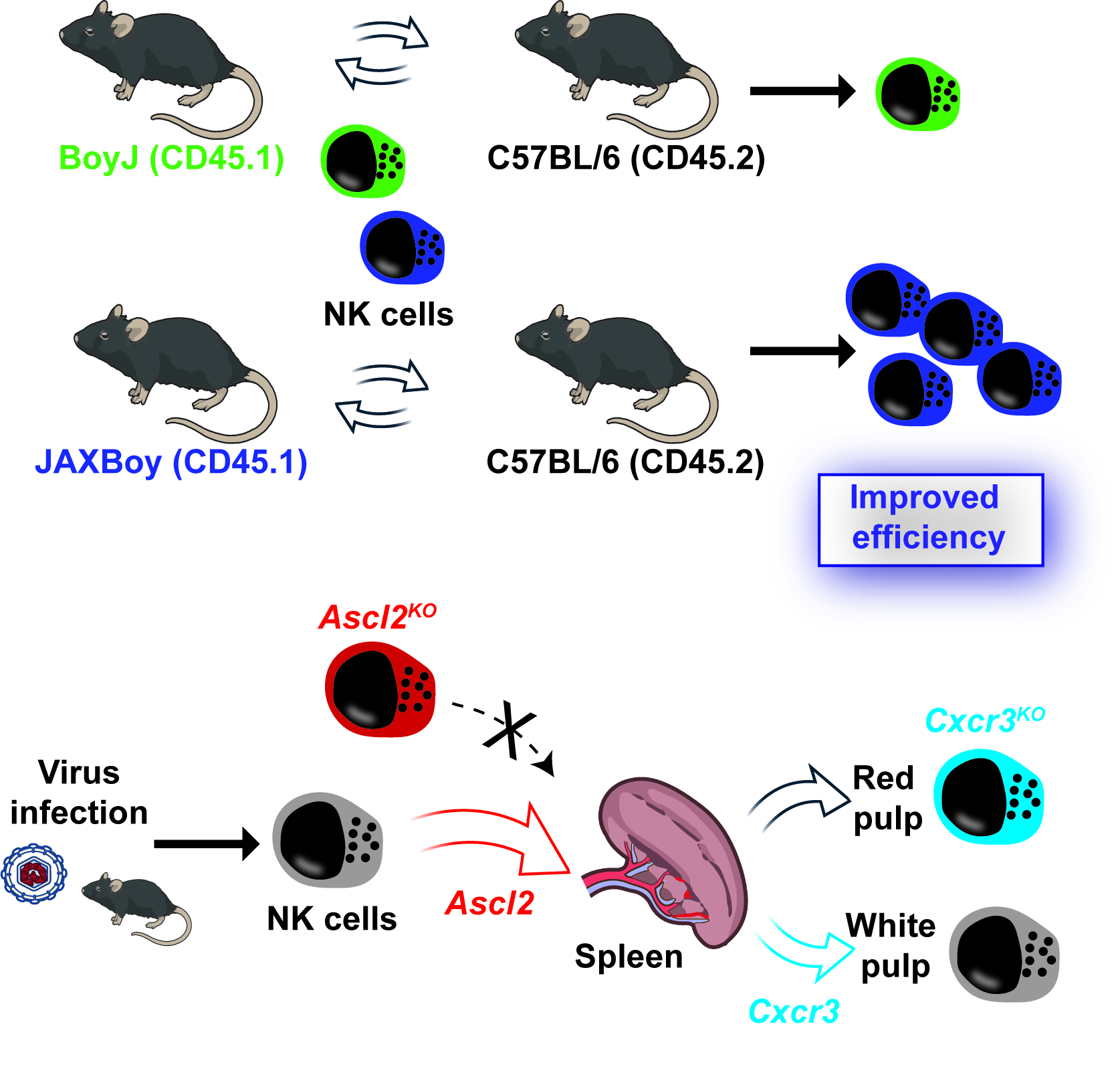

Supplement: vkaf122_Supplementary_Data [file vkaf122_supplementary_data.zip › JIMMUN-24-00315-s02.tif]
